# Intestinal mast cell-derived leukotrienes mediate anaphylactic response to ingested antigens

**DOI:** 10.1126/science.adp0246

**Published:** 2025-08-07

**Authors:** Nathaniel D. Bachtel, Jaime L. Cullen, Min Liu, Steven A. Erickson, Vassily I. Kutyavin, Darine W. El-Naccache, Esther B. Florsheim, Jaechul Lim, Zuri A. Sullivan, Raiden Imaeda, Andrew Hudak, Cuiling Zhang, Ruslan Medzhitov

**Affiliations:** 1 Department of Immunobiology, Yale University School of Medicine, New Haven, Connecticut 06520, USA.; 2 School of Life Sciences, Arizona State University, Tempe, Arizona 85287-7501, USA.; 3 College of Veterinary Medicine and Research Institute for Veterinary Science, Seoul National University, Seoul 08826, Republic of Korea.; 4 Department of Molecular and Cellular Biology, Harvard University, Cambridge, MA; 5 Howard Hughes Medical Institute.; 6 Tananbaum Center for Theoretical and Analytical Human Biology, Yale University School of Medicine, New Haven, CT, USA

## Abstract

Anaphylaxis is a life-threatening complication of food allergen exposure. While mechanisms governing anaphylaxis after intravenous injection are defined in mice, these models neglect mucosal exposure that accompanies ingestion. We investigated the role of mast cells within the intestine of mice in response to antigens delivered orally. Oral anaphylaxis required IgE-FcεR1 signaling, and profiling of intestinal mast cells revealed a rapidly developing population shaped by epithelial cues. Intestinal mast cells were largely epithelium-resident and displayed divergent transcriptomes and effector functions from connective tissue mast cells found throughout the body. Histamine synthesis was diminished and leukotriene generation enhanced. Mice genetically deficient in cysteinyl leukotriene synthesis, or those treated with the aLOX5 antagonist, Zileuton, were protected from oral antigen-induced responses whereas those elicited by intravenous injection were unaltered.

## INTRODUCTION

Food allergies are exaggerated immune responses against protein antigens found in food (1–5). These immune responses are thought to be predominately driven by IgE-mediated recognition of dietary antigens, leading to cross linking and degranulation of mast cells throughout tissues. In the most severe cases, food allergen exposure leads to anaphylaxis, a life-threatening condition characterized by bronchoconstriction, vascular leak, and hemodynamic collapse (6). While mast cell mediators that can drive anaphylaxis induced by intravenous allergen administration have been identified in mice, those capable of doing so secondary to ingestion remain unclear (7, 8). This suggests that mucosal exposure alters the requirements needed to produce anaphylactic responses and that identifying such pathways could offer treatment options for food allergies and related conditions.

Ingestion preferentially drives food antigens to the gastrointestinal lumen, where they must traverse the epithelial layer before being exposed to the mucosal immune system. This transport occurs predominantly in the proximal small intestine and is mediated by an active process involving IL13-mediated secretory cell antigen passages (SAPs) and increases in paracellular permeability (9). Studies utilizing IgE and FcεR1a-deficient strains, and those lacking the mast cell growth factor IL9, suggest local intestinal mast cells play crucial roles in driving this response (10–13). Mice overexpressing IL9 display enhanced intestinal mast cell expansion, epithelial barrier permeability, and susceptibility to oral anaphylaxis, whereas IL9/IL9R KO mice are resistant to oral, but not intravenous allergen challenge (14, 15). We reasoned that enhancing understanding about the development and function of intestinal mast cells could define mediators contributing to food-induced anaphylaxis.

We focused on cysteinyl leukotrienes (CysLTs) as potential candidates secreted by intestinal mast cells to augment oral anaphylaxis severity. CysLTs are eicosanoid lipid mediators that are produced by multiple cell types of hematopoietic and non-hematopoietic origin in response to diverse environmental signals (16). They are formed by the oxidation of arachidonic acid by 5-lipoxogenase (aLOX5) and accessory protein 5-lipoxogenase activating protein (FLAP), to leukotriene A4 (17–19). LTA4 is then conjugated to glutathione by the enzyme LTC4 synthase (LTC4S) to form LTC4 and exported from the cell (20, 21). Extracellular LTC4 is rapidly converted to LTD4 by γ-glutamyl transferase (GGT) and γ-glutamyl leukotrienase (GGL) and terminally to LTE4 by dipeptidase (DPEP) enzymes which are abundant in the intestinal epithelium (22, 23). Thus, changes in the synthesis and degradation of CysLTs in the intestine may be manipulated at multiple steps leading to excess. CysLTs mediate their effects through three receptors that differ in their relative affinities for each species: CysLTR1 binds predominantly to LTD4 with lesser affinity for LTC4 and LTE4, CysLTR2 with equal affinities for LTC4 and LTD4, and CysLTR3 (OXGR1) binds selectively to LTE4 (24–26). These receptors differ in their cellular expression but largely converge on activation of ILC2s (27–29). Given that ILC2s are potent sources of IL9 and IL13 which both sensitize to oral anaphylaxis, CysLT engagement could thus serve as a link between local intestinal responses and severe anaphylactic reactions (30–33).

## RESULTS

### Ingested allergen-induced anaphylaxis relies on IgE-FcεR1 signaling

**Fig 1.**
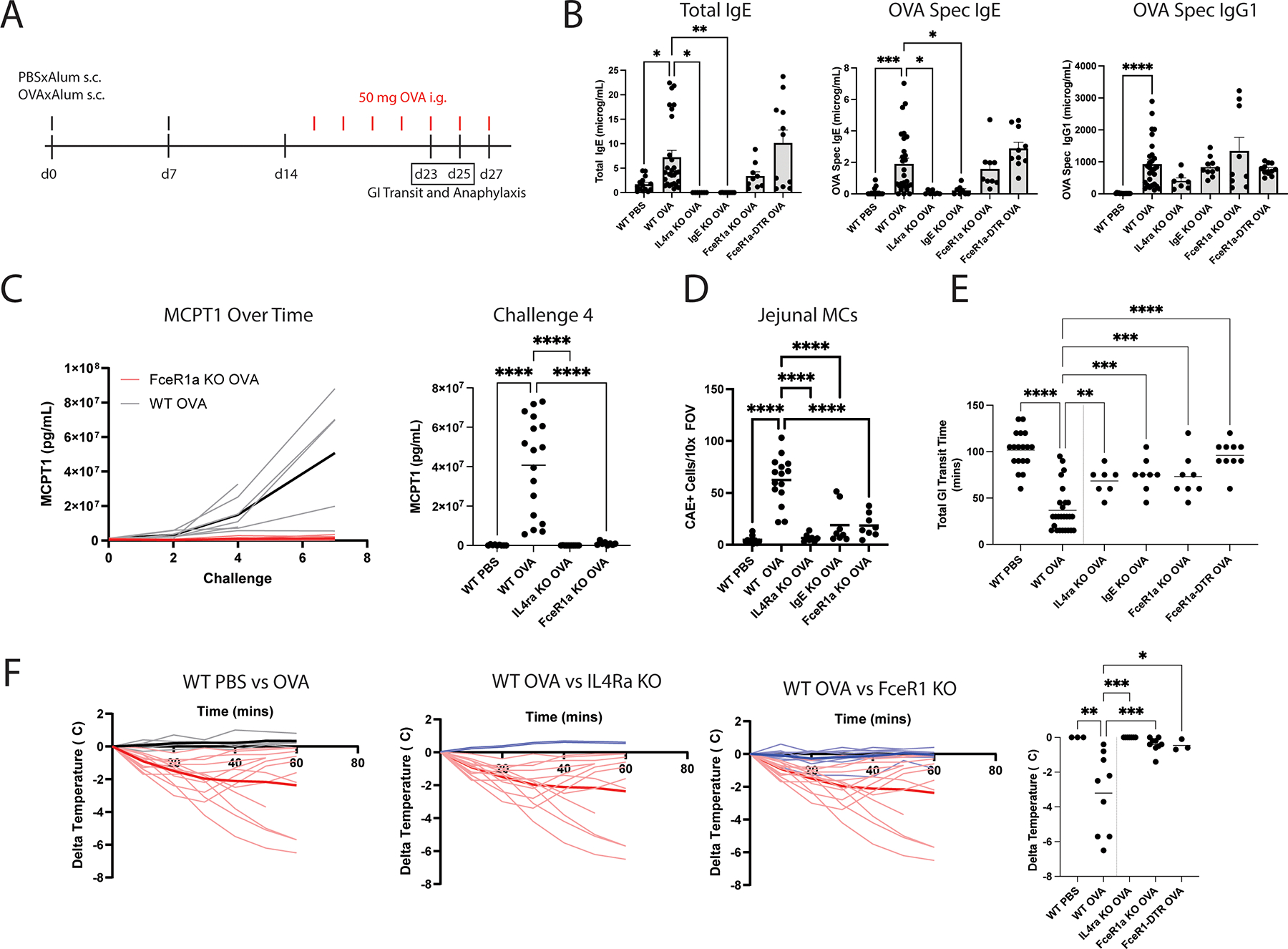
The IgE-FcεR1a axis is required for ingested food induced anaphylaxis. (A) Experimental scheme: WT BALB/cJ, IL4ra KO, IgE KO, FcεR1 KO, or FcεR1-DTR mice were sensitized and challenged with i.g. OVA (B) Serum levels of total IgE, OVA-specific IgE, and OVA-specific IgG1 antibodies at d27. (C) MCPT1 levels over time and at challenge 4 (D) quantification of CAE+ mast cells/10x jejunal FOV. (E) Total GITT of allergic or control mice at 5^th^ OVA challenge by carmine red total GI transit assay. (F) Temperature drop over time to 6^th^ i.g. OVA challenge (L) and (R) greatest temperature drop in 1hr. n=4–28 mice per group, pooled from at least 2 independents. Statistics were performed by one way ANOVA with multiple comparisons test. Each dot represents an individual mouse. Horizontal lines represent mean. *p<0.05, **p<0.01, ***p<0.001, and ****p<0.0001.

Prior studies report IgE-dependent requirements for oral anaphylaxis but differ in the susceptibility of mouse background strains and experimental food allergy paradigms used (34–36). We began by testing the OVA/alum model whereby BALB/cJ mice are sensitized subcutaneously two times with PBS or OVA adsorbed in aluminum hydrogel and subsequently boosted by multiple intragastric challenges delivered by oral gavage. Sensitization elicits IgE-class switching and production of antigen-specific IgE by B cells, whereas boosting activates and recruits intestinal immune cells, drives IL9-mediated intestinal mast cell expansion, and ultimately results in oral anaphylaxis susceptibility. Allergen-inducible gastrointestinal transit time (GITT) was measured at challenge 5 by administering a mixture of food allergen and carmine red dye and quantifying time until red stool was observed in the cage; oral anaphylaxis was assessed at challenge 6 by rectal thermometry (Fig 1A, Fig S1A). Given that IL4 is necessary for B cells to class switch to IgE antibodies, and FcεR1a binds to IgE on the surface of mast cells and basophils, IL4Ra KO, IgE KO, and FcεR1a KO mice were utilized to determine whether the responses observed were attributable to IgE-FcεR1a signaling (37, 38). Finally, FcεR1a-DTR mice, which express diphtheria toxin receptor under the FcεR1a locus, were utilized to conditionally ablate mast cells and basophils upon administration of diphtheria toxin (39).

Quantification of total and OVA-specific IgE and IgG1 antibodies confirmed IgE production was absent from IL4Ra KO and IgE KO mice, whereas it remained unaltered in FcεR1a KO or DT-treated FcεR1a -DTR mice (Fig 1B). OVA-specific IgG1 antibodies were present in all strains. Serial blood draws taken 1 hour after every other challenge revealed escalating mast cell protease 1 (MCPT1) serum levels in sensitized mice which were absent in FcεR1a KO and IL4Ra KO mice, highlighting increased levels of IgE-mediated mast cell degranulation (Fig 1C). Indeed, expansion of mucosal mast cells, as determined by chloroacetate esterase (CAE) staining of jejunal segments, was notable in WT mice but reduced in those deficient in IgE-FcεR1a signaling (Fig 1D). This suggested mast cell degranulation initiated local expansion in this experimental paradigm. While none of the strains tested displayed altered baseline GITT (Fig S1B), each displayed a ~25% reduction in GITT upon oral allergen administration as compared to ~75% in WT mice (Fig 1E, Fig S1C) and all displayed ~85–100% less severe hypothermic responses elicited by oral challenge (Fig 1F). Thus, cardinal features of anaphylaxis induced by oral antigens, including diarrhea and allergen-induced temperature drop in this model, are strongly reliant on IgE-FcεR1a signaling.

We next investigated whether anaphylaxis induced by intravenous injection was dependent on IgE-FcεR1a signaling as well (Fig S1D). BALB/cJ mice were sensitized with PBSxAlum or OVAxAlum, but then challenged with OVA intravenously rather than intragastrically on day 14. We observed no requirement for either FcεR1a-expressing cells (Fig S1E), or IgE (Fig S1F) upon intravenous allergen challenge, suggesting major contributions of IgG1-induced anaphylaxis at the doses utilized (40–42).

Given that oral challenge elicited anaphylactic symptoms, we sought to examine the degree of mast cell activation across tissues. We utilized staining for toluidine blue (TB), which labels mast cell metachromatic granules and enables identification of degranulated mast cells by poor intracytoplasmic staining or elevated extracellular granule release (43).

We compared degranulated MCs in the ear pinna, stomach, and bronchus induced after the 6^th^ oral challenge (Fig S1G) with those induced by passive IgE-dependent anaphylaxis, whereby mice are primed systemically with antigen-specific IgE and challenged intravenously with antigen (Fig S1H). We observed mast cell degranulation in all tissues examined after oral challenge, although the percentage of degranulated cells was at-most half of that observed upon intravenous injection (Fig S1I–J). Thus, oral anaphylaxis represents a state of mild, but not absent, systemic engagement of mast cells.

We utilized the peanut/cholera toxin model (35) in BALB/cJ mice to determine whether the same requirements for oral anaphylaxis could be generalized to other allergens and adjuvants (Fig S1K). Oral peanut challenge induced no temperature drop (Fig S1L); however, a response to intravenous challenge with crude peanut extract was detectable (Fig S1M–N), suggesting that differences between mouse colonies, background strains, or pathogen burden may influence the effectiveness of the peanut/cholera toxin model in driving oral allergen-induced anaphylaxis.

From our data, we concluded that oral anaphylaxis represents a state of mild systemic and strong local mast cell activation that is reliant on IgE-FcεR1a signaling.

### Intestinal mast cells are a rapidly developing population shaped by epithelial TGFb

Mast cells comprise two distinct lineages, connective tissue mast cells (CTMCs) and mucosal mast cells (MMC), which display different developmental trajectories, tissue localizations, and functions (44). Connective tissue mast cells express high levels of negatively charged proteoglycans that can be stained using conjugated avidin, and are located near the muscularis layer of the intestine. In contrast, mucosal mast cells stain poorly with avidin, but positively for MCPT1, and are found in the lamina propria and epithelial layers (45). MMCs can be distinguished from CTMCs by lack of expression of the Mrgprb2 receptor and their differing expression of mucosal and epithelium-homing integrins (46, 47). Given that CTMCs are seeded during embryonic development, they are negative for integrin b7 whereas mast cell progenitors require expression of the integrin a4b7, and extravasate into the intestine by interacting with MADCAM-1 (48, 49). Mucosal mast cell progenitors proliferate in the lamina propria, and gradually decrease a4 and increase CD103 (ITGAE) as they home to the epithelium (50).

**Fig 2.**
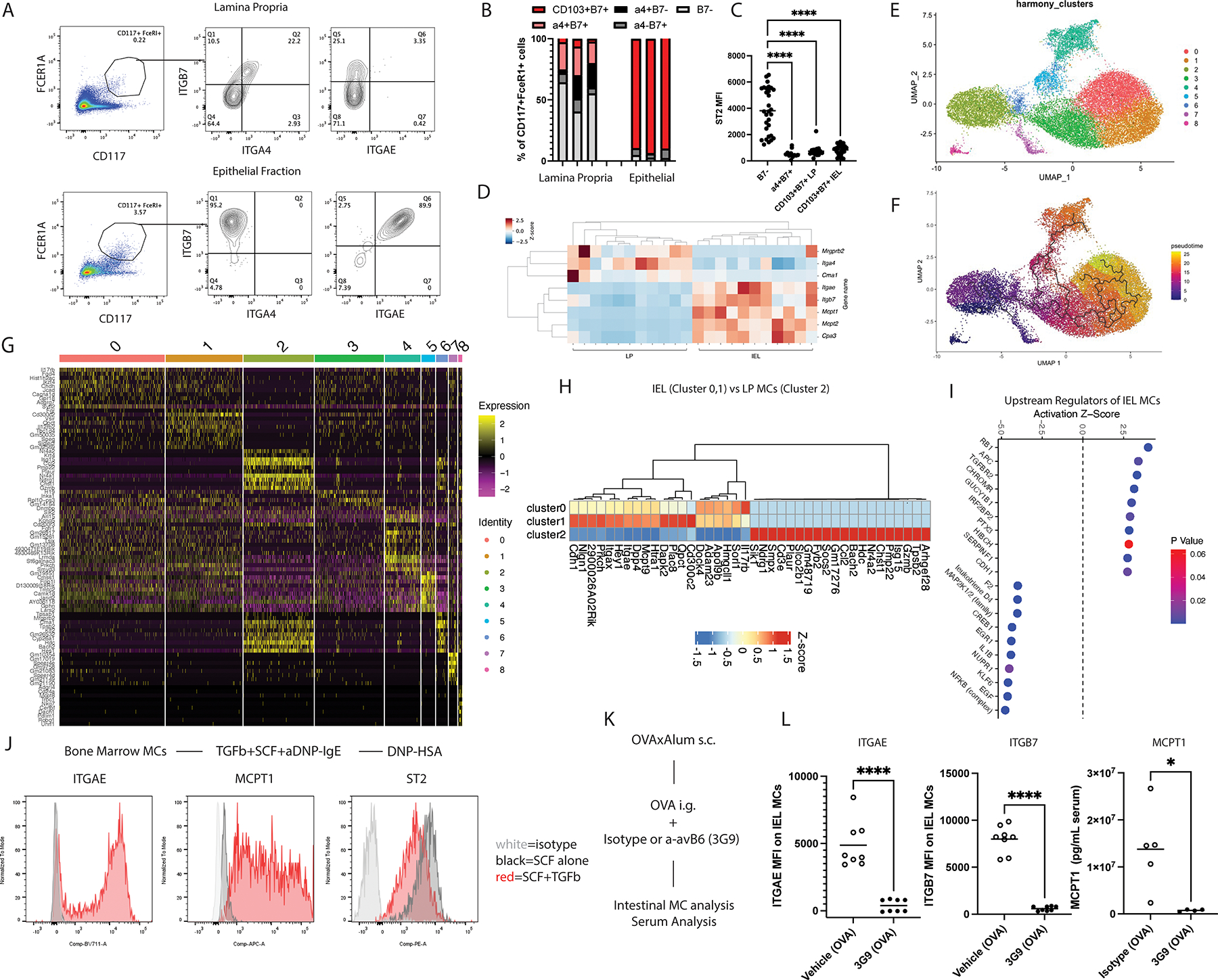
Intestinal mast cells are imprinted by epithelial cues. (A) Staining of CD117 and FcεR1 in lamina propria and intraepithelial immune cells from naïve BALB/cJ mouse small intestine with ITGB7, ITGA4, and ITGAE staining (Fig S2). (B) Percentage of b7-, a4b7+, and CD103b7+ mast cells in intraepithelial and lamina propria fractions (each bar represents a single naïve mouse) and (C) ST2 MFI of mast cell subsets with different integrin staining. RNAseq was performed on sorted intraepithelial or lamina propria mast cells from BALB/cJ mice sensitized and challenged once with 20mg of OVA i.g. (D) Heatmap of mast cell proteases and integrins between sorted populations (E) scRNAseq clusters of intestinal mast cells sorted from sensitized BALB/cJ mice challenged five times with 50mg OVA i.g. (22171 cells were analyzed) (F) pseudotime analysis of intestinal mast cell development starting from *Mcpt8*+*Itga4*+ mast cell precursors. (G) heat map of top 10 DEGs between each mast cell cluster (H) heat map of top DEGs between IEL mast cells (clusters 0,1) and LP mast cells (cluster 2) (I) IPA analysis of these DEGs reveals predicted upstream regulators (J) BMMCs were treated with TGFB+SCF for 7 days and effects on epithelial mast cell shaping were quantified by flow cytometry (K) BALB/cJ female mice were sensitized as previously described and challenged with OVA in conjunction with isotype or avb6 neutralizing antibody (3G9) treatment and (L) expression of ITGAE and ITGB7 on intestinal mast cells, and serum MCPT1 post challenge were quantified. n=3–30 mice per group. All data was pooled from at least 2 independent experiments except for scRNAseq data which was obtained in one. Each box in heatmap in D represents mast cells sorted from a single mouse. scRNAseq plots E-I represent data from IEL and LP mast cells sorted from two mice, sequenced separately, and concatenated for final analysis. Statistics were performed by one way ANOVA with multiple comparisons test (C), or unpaired t-test (L). Each dot represents an individual mouse. Horizontal lines represent mean. *p<0.05, **p<0.01, ***p<0.001, and ****p<0.0001.

We performed flow cytometry on the small intestine of naïve BALB/cJ mice to determine the composition of intestinal mast cell populations under homeostatic conditions. We hypothesized that intestinal mast cells may express molecules involved in oral anaphylactic responses (Fig S2). We observed that lamina propria (LP) mast cells constitute approximately 0.01–0.02% of lineage-negative CD45+ cells, whereas they comprise 0.025–0.05% of the epithelial (IEL) fraction (Fig 2A). Within the lamina propria, we observed three distinct mast cell populations based on integrin staining. Approximately 50–60% of mast cells were negative for b7 integrin, suggestive of a sizable population of connective tissue mast cells, with 20–40% positive for a4 and b7, and 10–20% positive for CD103 and b7, but lacking a4 expression. The epithelial population was almost entirely composed of CD103/b7^+^ mast cells (85–95%), suggestive of terminally differentiated mast cells attached to the epithelial layer (Fig 2B). Besides integrin expression, these mast cells differed in the expression of canonical mast cell surface markers, with a4b7^+^ and CD103b7^+^ mast cells expressing a lower MFI of ST2 relative to b7^−^ mast cells (Fig 2C).

To understand these populations’ functions and development, we sorted intraepithelial and lamina propria mast cells from WT BALB/cJ mice that had been sensitized and challenged once orally and performed RNAseq. The primary differences observed were between mast cells sorted from different compartments of the small intestine (Fig S3A–B, Table S1). Sorted epithelial mast cells were enriched in transcripts for *Mcpt1* and *Itgae*, whereas they were relatively deficient for *Mrgprb2* and *Cma1* expression, in line with our prior observations regarding localization of connective tissue and mucosal mast cells between these sites (Fig 2D, Fig S3C–E).

B cell and epithelial cell transcripts were also observed as being differentially expressed between sorted IEL and LP mast cell populations, suggestive of minor cellular contamination introduced during sorting. Therefore, we performed scRNAseq of mast cells sorted from mice sensitized and challenged 5 times with oral allergen (Fig S3F, Table S2). With non-mast cells removed from the dataset, we found transcriptional differences between mast cells found in LP and IEL sorted samples (Fig S3G). Unbiased harmony clustering revealed 9 independent clusters all of which expressed *Kit* and *FcεR1a* (Fig 2E, Fig S3H–I, Table S3)(51). Pseudotime analysis, beginning from the *Itga4*+ *Mcpt8*+ mast cell progenitor cluster 8, revealed a developmental spectrum from cluster 2 LP cells, which closely resembled connective tissue mast cells but largely did not express *Mrgprb2*, and ending with clusters 0, 1 which were found dominantly in the epithelial layer (Fig 2F–G, Fig S3J–M)(52). Given the substantial transcriptional overlap between *Mrgpr2b*+ mast cells and cluster 2, we performed DEG analysis to identify genes most associated with the acquisition of the epithelial mast cell phenotype by comparing this cluster with clusters 0,1 (Fig 2H, Fig S3N, Table S4). The top DEGs increased in expression in IEL MCs were molecules involved in cell adhesion (*Cdh1)*, integrins (*Itgax, Itgae*), incretin metabolism (*Dpp4*), synapse formation *(Nlgn2),* cytokine signaling (IL25 and IL17b receptor, *Il17rb*) and notch signaling (*Hey1*), whereas expression of *Tpsb2, Ndrg1,* and *Plaur* were strongly suppressed (Fig 2H, Fig S3O, Table S5). Many of these genes overlapped with those defined in a recent study in helminth infection, suggesting a conserved terminal phenotype expanded in states of type 2 inflammation (53).

We used Ingenuity Pathway Analysis (IPA) to predict upstream regulators of the epithelial mast cell phenotype, of which TGFb was the strongest predicted extracellular signal regulating epithelial mast cell development (Fig 2I, Fig S3P, Table S6). To determine whether TGFb was capable of mimicking the transcriptional and functional changes we observed between mast cell subsets, we cultured BALB/cJ female bone marrow-derived mast cells with the mast cell survival factor, stem cell factor (SCF), in the presence or absence of TGFb for 7 days (Fig S4A)(52). TGFb treatment markedly enhanced the cell surface expression of CD103 and MCPT1, as measured by flow cytometry, while downregulating ST2 (Fig 2J, Fig S4B–C).

Prior studies have suggested epithelial TGFb, which is liberated by the avb6 integrin, drives mucosal mast cell protease expression (54, 55). To determine whether avb6 may similarly underly the acquisition of the epithelial mast cell phenotype in the intestines of food allergic mice, we sensitized BALB/cJ mice with PBSxAlum or OVAxAlum subcutaneously and subsequently treated them with isotype or avb6 neutralizing antibody (3G9) throughout the oral challenge phase (Fig 2K, Fig S4F)(56). While 3G9 treatment did not alter mast cell expansion and localization to the epithelial layer (Fig S4G), it reduced mast cell expression of ITGB7 and CD103, while increasing ST2 (Fig S4H–I). Furthermore, MCPT1 levels were suppressed in sensitized 3G9-treated mice despite an increased frequency of IEL mast cells relative to isotype treated mice (Fig 2L, Fig S4J). Effects on oral anaphylactic responses were less clear, with one experiment suggesting suppression and another displaying modest or no effect between groups (Fig S4K). We concluded that intestinal mast cells were a rapidly developing mucosal population that differentiate in response to avb6-mediated epithelial TGFb release.

### Intestinal mast cell differentiation skews mediator production

Intravenous injection of allergens in mice drives anaphylaxis through *Mrgprb2*+ connective tissue mast cells and the release of histamine (57, 58). These *Mrgprb2*+ mast cells are located throughout the body but are the considerable minority at mucosal sites such as the lung and intestine. Given that oral challenge reflected a state of high-local and low-systemic mast cell activation, we hypothesized that shaping of mast cells in the intestines may lead to altered histaminergic requirements for responses originating from ingestion.

**Fig 3.**
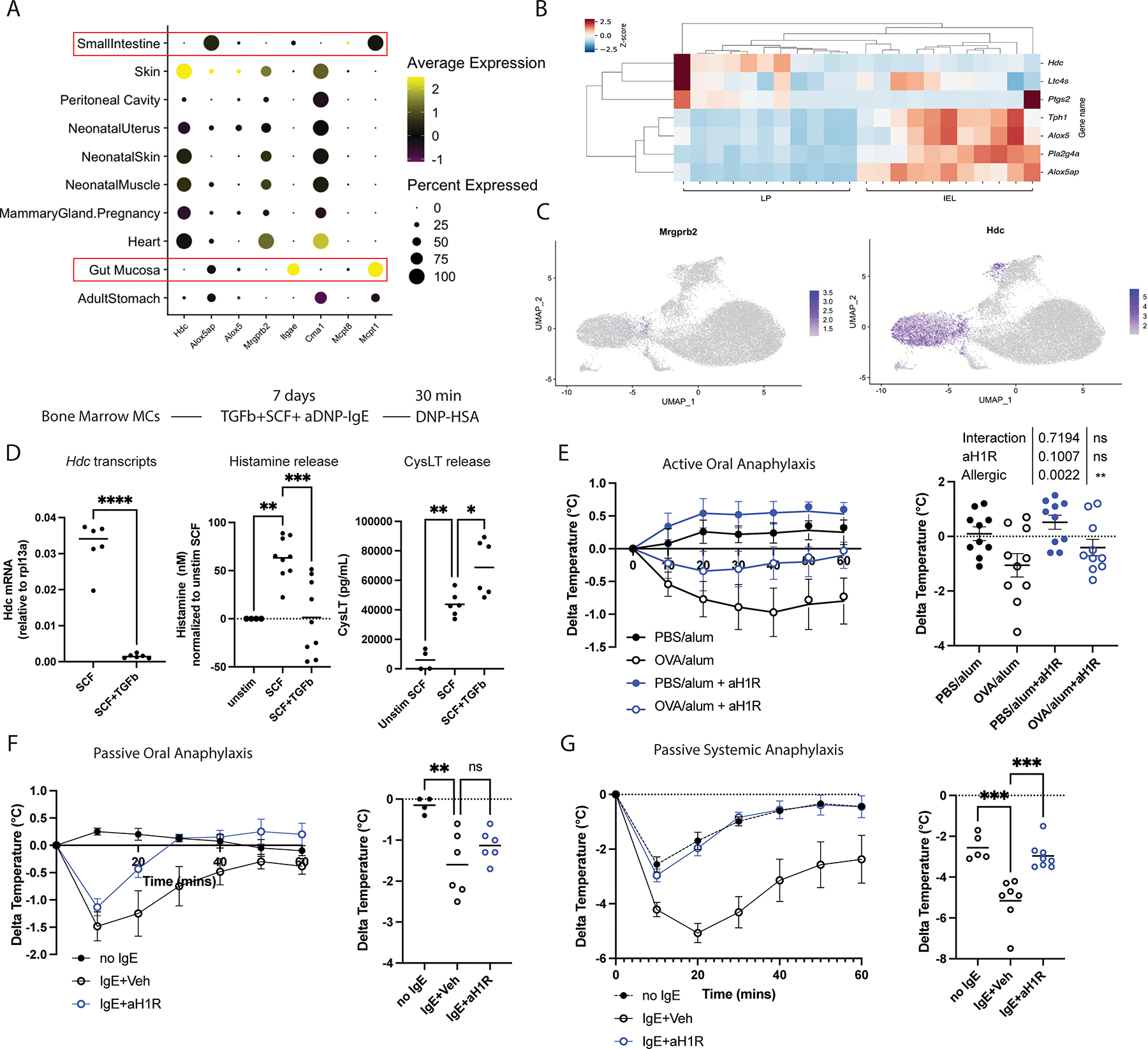
Mucosal imprinting skews mediator production and route-dependent anaphylaxis requirements. (A) Dot plot of key mast cell marker and effector genes across tissues (59) (B) heat map of mast cell mediators differentially expressed by IEL and LP mast cells by bulk RNAseq (C) UMAP plot demonstrating minimal *Mrgprb2*+ mast cells and loss of *Hdc* with intestinal mast cell development (22171 cells were analyzed) (D) *Hdc* transcripts and Histamine and CysLT release by ELISA of BMMCs treated with SCF+TGFb for 7 days (E) Effects of H1R antagonism on severity of active oral anaphylaxis in BALB/cJ mice was assessed by means of triprolidine pre-treatment. (F) Effects of H1R antagonism on passive oral anaphylaxis and (G) passive systemic anaphylaxis was similarly examined. n=4–10 mice per group, pooled from at least 2 independent experiments. n=4–9 technical replicates from 3 independent experiments for D. Each box in heatmap in b represents mast cells sorted from a single mouse. scRNAseq plot c represent data from IEL and LP mast cells sorted from two mice, sequenced separately, and concatenated for final analysis. Statistics were performed by unpaired t-test in (D) – left panel; one-way ANOVA with multiple comparisons in (D) middle and right panels and (F) and (G); and two-way ANOVA in (E). Each box of heatmap in (B) represents mast cells sorted from a single mouse. Each dot represents an individual mouse. Horizontal lines represent mean. *p<0.05, **p<0.01, ***p<0.001, and ****p<0.0001.

Utilizing a recently published multi-tissue mast cell scRNAseq atlas, we compared marker genes of key clusters identified in the intestine to other tissues throughout the body (Fig 3A)(59). *Mcpt1* and *Itgae* were only observed in mast cells originating from the gastrointestinal tract, whereas *Mrgprb2* and a connective tissue mast cell associated protease, *Cma1,* were dominant in nearly all other tissues tested. Expression of histidine decarboxylase (*Hdc)*, the enzyme required for mast cell histamine synthesis, was associated with the connective tissue phenotype and minimally expressed in the intestine, whereas 5-LOX activating protein (*Alox5ap)*, an enzyme necessary for leukotriene production, was more prevalent in intestinal mast cells.

Examination of our intestine-intrinsic sequencing datasets revealed that not all intestinal mast cells are deficient in *Hdc*. Those isolated from the lamina propria were histamine rich relative to the dominant population found in the intraepithelial layer (Figure 3B, Fig S3E) and this was not due to the presence of *Mrgprb2*+ connective tissue mast cells at this site (Figure 3C). Rather, intestinal mast cells transition through a transient transcriptional state similar to that of connective tissue mast cells (including expression of *Hdc* and *Cma1*) after entering the intestine, which was altered as they localize to the epithelium and terminally differentiate.

As TGFb was sufficient to drive gene expression of bone marrow mast cells towards an epithelial mast cell phenotype, we tested the effect of TGFb treatment on transcription and the release of select mediators. TGFb treatment decreased *Hdc* mRNA by ~20 fold. Genes involved in leukotriene generation were also changed in expression, with *Alox5ap* being upregulated ~2-fold by TGFb, and *Ltc4s* being downregulated (Fig 3D, Fig S4D). Histamine (Fig. 3D) and prostaglandin D2 (PGD2) (Fig S4E) release were suppressed following degranulation induced by aDNP-IgE and DNP-HSA treatment, whereas MCPT1 (Fig S4E) and cysteinyl leukotriene synthesis (Fig. 3D) were enhanced.

Histamine mediates its effect on IgE-dependent anaphylaxis to intravenous allergens dominantly through histamine receptor 1 (H1R), which can be blocked by acute pharmacological antagonism with Triprolidine (60, 61). Administration of Triprolidine thirty minutes before oral challenge did not block a drop in body temperature (Figure 3E); however, the percentage of mice that dropped greater than two degrees in body temperature was reduced. Similarly, Triprolidine pretreatment had a non-significant effect on anaphylaxis elicited by passively priming mice with TNP-IgE and subsequently challenging intragastrically with TNP-OVA (Fig 3F).

The results observed could not be due to ineffectiveness in dosing, timing or pharmacological properties of Triprolidine itself, as mice passively sensitized with IgE and challenged intravenously were nearly completely protected (Fig 3G). We therefore conclude that a local non-histaminergic response mediated by intestinal mast cells precedes and, in some cases, sensitizes to more severe reactions that follow.

### Hematopoietic cysteinyl leukotriene are required for the acute oral anaphylaxis response.

**Fig 4.**
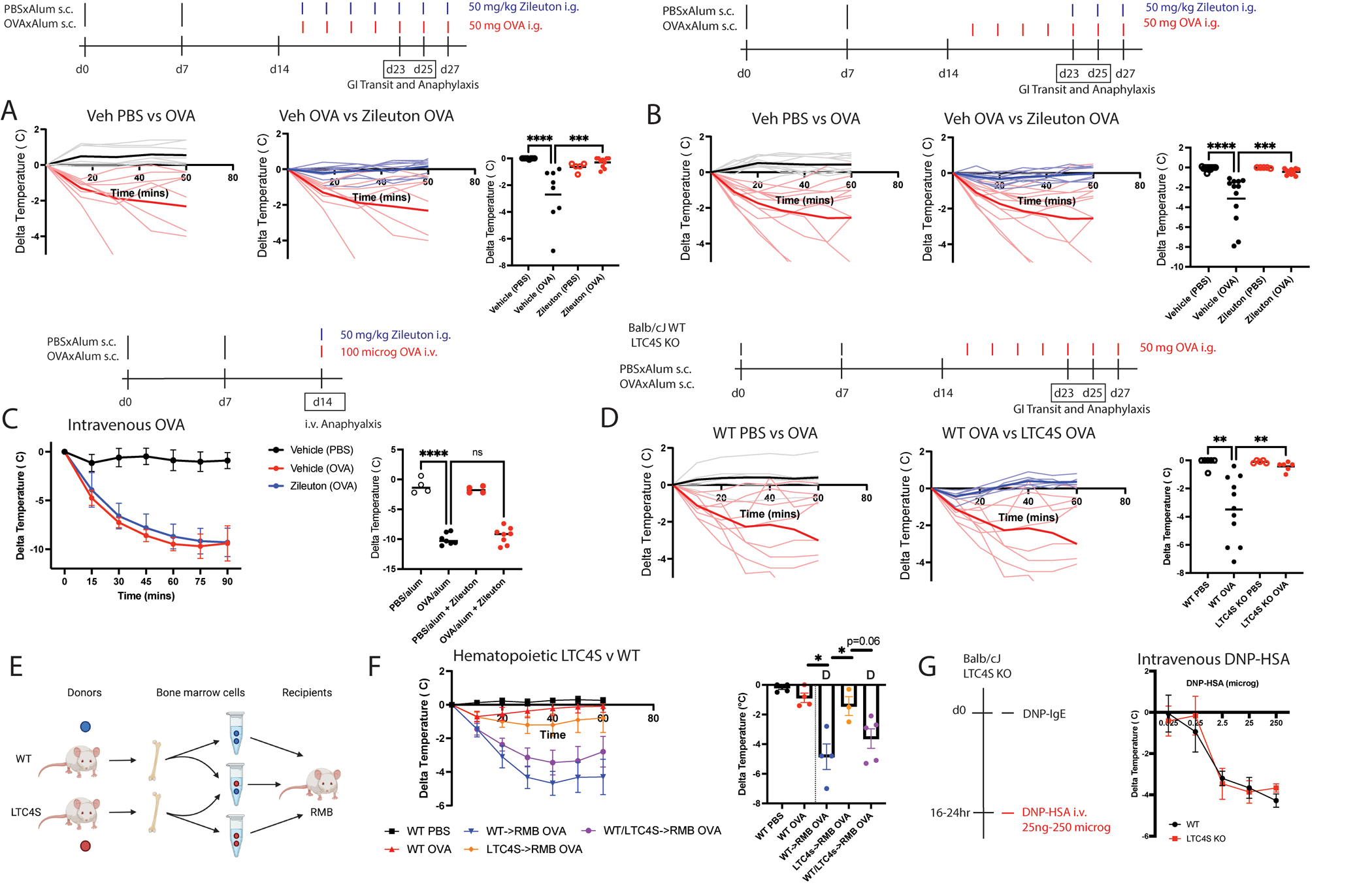
Hematopoietic cysteinyl leukotriene production constitutes an acutely inhibitable pathway required for oral anaphylaxis. (A) Effect of chronic Zileuton administration on severity of active oral anaphylaxis in BALB/cJ (B) effects of acute Zileuton administration from 5^th^ challenge on severity of oral anaphylaxis in BALB/cJ (C) effects of acute Zileuton administration on severity of intravenous anaphylaxis (D) effect of LTC4S KO or WT BALB/cJ status on severity of active oral anaphylaxis (E) FcεR1-DTR (RMB) mice were sublethally irradiated and transplanted with bone marrow cells derived from WT or LTC4S KO mice, or a mix of 1:1 WT and LTC4S KO mice. The mice were then rested for 8 weeks before being sensitized and orally challenged as previously described. (F) Temperature drop over time after 4^th^ oral OVA challenge and greatest temperature drop. D denoted death within a mouse group. (G) LTC4S KO mice were passively sensitized with aDNP-IgE and challenged 16–24 hours later with 25ng-250ug of DNP-HSA intravenously and anaphylaxis severity assessed by greatest rectal temperature over the ensuing hour. n=3–11 per group for mouse experiments across 2–3 independent experiments except for panels E-F which are from one. Statistics were performed by one-way ANOVA with multiple comparisons in A-D, G and unpaired t-test in F. Each dot represents an individual mouse. Horizontal lines represent mean. *p<0.05, **p<0.01, ***p<0.001, and ****p<0.0001.

We focused on leukotrienes as non-histamine molecules that could be important in gastrointestinal allergic reactions because enzymes required for leukotriene biosynthesis and its release were enhanced by intestinal mast cell differentiation. To examine this possibility, we sensitized BALB/cJ mice with PBSxAlum or OVAxAlum and treated them with the aLOX5 antagonist Zileuton throughout the oral challenge phase (Fig 4A, Fig S5A). Zileuton-treated mice displayed no alterations in levels of allergen-specific IgE or IgG1 antibodies (Fig S5B) but exhibited reduced mast cell expansion and degranulation (Fig S5C–D) relative to vehicle-treated controls. Zileuton-treated mice did not show a decrease in body temperature (Fig 4A) nor a decrease in gastrointestinal transit time (Fig S5E) in response to oral challenge with OVA. We repeated the experiment but treated with Zileuton only only 1 hour prior to GITT evaluation instead of throughout the boosting-phase (Fig 4B, Fig S5F). Intestinal mast cell expansion was not different between vehicle or Zileuton-treated mice, but MCPT1 levels were reduced by half (Figure S5G–H). Acute Zileuton treatment prevented oral anaphylactic temperature drop (Fig 4B) and also ameliorated allergen-induced reductions in GI transit time to ~1/3 of sensitized vehicle treated levels (Fig S5I).

We considered that Zileuton may be able to inhibit allergen-inducible changes in intestinal permeability which could explain such an acute effect, but did not find evidence of changes in uptake of 4kD or 70kD dextrans when co-gavaged with allergen in food-allergic mice (Fig S5J). Systemic mast cell degranulation, as measured by toluidine blue staining of ear pinna, stomach, and bronchus one hour after oral challenge, was also not reduced upon acute Zileuton pre-treatment (Fig S5K). Furthermore, treatment of bone marrow mast cells *in vitro* with Zileuton led only to minor reductions in degranulation as measured by beta-hexosaminidase release (Fig S5L–M).

To investigate whether activity of aLOX5 in the gut mucosa was required for anaphylaxis, we sensitized BALB/cJ mice with PBSxAlum or OVAxAlum as previously described and pretreated them with Zileuton one hour prior to intravenous allergen administration (Fig 4C). We also compared C57BL/6J aLOX5 KO mice with C57BL/6J littermate controls following sensitization and intravenous challenge (Fig S5N). Blocking or deficiency in aLOX5 did not impact the decrease in body temperature following systemic antigen challenge nor impact symptoms of anaphylaxis (Fig 4C and Fig S5O–P); however, an effect on hemoconcentration was observed by measurement of blood hematocrit levels (Fig S5Q). We concluded that leukotrienes mediated anaphylactic response to oral antigens, but this was not dependent on altered intestinal permeability or systemic mast cell activation.

To determine whether the effects of Zileuton were attributable to the actions of cysteinyl leukotrienes, we sensitized and challenged LTC4S KO mice or WT littermates with oral allergen (Fig 4D, Fig S5R). No differences were observed in antigen-specific IgE or IgG1 levels, although LTC4S KO mice displayed a mildly elevated level of total IgE relative to WT controls (Fig S5S). Mucosal mast cells were unchanged in number; however, serum levels of MCPT1 induced by oral challenge were reduced approximately by half (Fig S5T–U). LTC4S KOs were partially protected from allergen-inducible reductions in GI transit time and were completely protected from oral anaphylactic temperature drops (Fig 4D, Fig S5V).

Cysteinyl leukotrienes can be produced by hematopoietic and non-hematopoietic sources, including tuft cells (62, 63). We sought to examine whether deficiency in the hematopoietic compartment would protect from oral anaphylaxis. We utilized a mixed bone marrow chimera model whereby WT, LTC4S KO, or a 1:1 mixture of WT and LTC4S KO bone marrow was transplanted into sublethally irradiated FcεR1-DTR (RMB) mice (Fig 4E). After waiting 8 weeks for bone marrow reconstitution, these mice were sensitized and orally challenged as described in Fig 4D. The body temperature of non-irradiated control mice dropped less than a degree at the 4^th^ oral challenge whereas mice which had WT bone marrow transplanted into RMB recipients (WT->RMB) dropped an average of 5 degrees in body temperature (Fig 4F). This increased hypothermic response was associated with an early rapid rise in MCPT1 serum levels at challenge 2 in all irradiated chimeric mice that was delayed in non-irradiated controls (Fig S5W). This suggests that some aspect of irradiation and bone marrow reconstitution may have sensitized to oral challenge and increased intestinal mast cell activity. The magnitude of body temperature drop in mice which were transplanted with LTC4S KO bone marrow (LTC4S->RMB) was reduced, whereas irradiated mice transplanted with a 1:1 mixture of WT and LTC4S KO bone marrow (WT/LTC4S->RMB) displayed no difference relative to the WT->RMB group. MCPT1 serum levels were generally associated with the protection observed. We, thus, concluded that cysteinyl leukotrienes originating from the hematopoietic compartment were required for ingested allergen-induced anaphylaxis.

The severity of the hypothermic response upon oral challenge in the OVA/alum model could be influenced by the effects of leukotrienes on both mast cell expansion (secondary to repeated intragastric challenges) and roles in the anaphylactic event itself. We therefore used passive models during which mice are administered antigen-specific IgE and subsequently challenged with antigen to elucidate how leukotrienes could influence events not attributable to mast cell expansion (Fig S6A). We utilized a model whereby mice were passively sensitized with aDNP-IgE and challenged intravenously with DNP-HSA. One week later, the same mice were passively sensitized with aTNP-IgE and challenged with TNP-OVA intragastrically (64–66). Intestinal mast cells were quantified one week after intragastric challenge, and blood was collected an hour after each challenge to measure mast cell degranulation. Both antigen-IgE clone combinations (aDNP-IgE, DNP-HSA and aTNP-IgE, TNP-OVA) elicited comparable hypothermic responses when antigen was administered intravenously, and the use of two clones of differing specificities prevented cross-reactivity between challenges (Fig S6B). These features enabled comparison of hypothermic responses elicited by IgE-mediated intravenous challenge to intragastric challenge in the same mice.

IgE-dependent anaphylaxis induced by intravenous antigen administration was largely unaltered by deficiency in CysLT function, either by genetic knockout of LTC4S, or loss of either of the receptors CysLTR1 or CysLTR2 (Fig S6C), with potential effects of LTC4S KO on hematocrit elevations seen at the dose administered (Fig S6D). Intragastric challenge led to a much milder hypothermic response that was completely dependent on LTC4S and CysLTR1 for its induction (Fig S6E), with a role for LTC4S on hematocrit elevations also observed (Fig S6F). MCPT1 quantified one hour after each challenge was increased in LTC4S KO and CysLTR2 KO strains, and intestinal mast cells detectible in the IEL and LP were unaltered in frequency as compared to WT controls (Fig S6G–H). Thus, CysLTs and CysLTR1 display elevated importance during IgE-dependent reactions occurring from gastrointestinal exposure and these effects do not appear attributable to decreased intestinal mast cells frequencies nor degranulation.

The selective role for leukotrienes in gastrointestinal challenge could reflect differences in the importance of these mediators at different concentrations of allergen that reach the bloodstream. To examine this possibility, we passively sensitized LTC4S KO or WT mice with aDNP-IgE and subsequently challenged them with DNP-HSA at doses ranging from 25 ng to 250 micrograms, encompassing systemic doses of allergen described after oral challenge (Fig 4G, Fig S6I). At each allergen concentration administered intravenously, there was no effect of LTC4S deficiency on body temperature responses (Fig S6J–K), and we, therefore, concluded that the requirement of CysLTs in oral anaphylaxis was tissue-intrinsic.

### Cysteinyl leukotriene receptor 1 and 2 are individually required for mucosal mast cell expansion, but do not acutely influence oral anaphylaxis.

To determine which cysteinyl leukotriene receptors were required for the effects on food allergen-induced diarrhea and oral anaphylactic temperature decreases, we sensitized and challenged WT, CysLTR1 KO, and CysLTR2 KO mice (Fig S7A). We observed no effect on OVA-specific or total IgE antibodies or OVA-specific IgG1 antibodies between these mice (Fig S7B). However, we observed a substantial decrease in MCPT1 in the blood that was similar to that observed in LTC4S KO mice (Fig S7C). Both CysLTR1 KO mice and CysLTR2 KO mice displayed a pronounced reduction in CAE+ jejunal mast cells (Fig S7D) and were protected from allergen-induced reductions in GI transit time (Fig S7E). In the absence of allergen challenge, CysLTR1 KOs had decreased GI transit times relative to WT controls.

While both strains displayed attenuated temperature drops from oral anaphylaxis relative to wild-type mice, CysLTR1 KOs had half the temperature drop of CysLTR2 KOs, suggesting a contribution of both receptors with a predominant effect of CysLTR1 (Fig S7F). Treatment with the CysLTR1 antagonist, Montelukast, prior to each oral challenge, also prevented body temperature changes in response to antigen and reduced intestinal mast cell expansion, while treatment with the CysLTR2 antagonist, HAMI3379, led to variable effects relative to CysLTR2 KOs (Fig S8A–G). Acute administration of Montelukast one hour prior to oral challenges 5–6 in PBSxAlum or OVAxAlum-sensitized BALB/cJ mice afforded no protection from oral anaphylaxis, unlike Zileuton administration (Fig S8H–N). This data would suggest an acute multi-receptor requirement in mediating anaphylaxis following active sensitization and challenge whereas CysLTR1 and CysLTR2 function non-redundantly to drive mucosal mast cell expansion.

## DISCUSSION

Although some individuals sensitized to food antigens develop anaphylaxis upon ingestion, others do not. While intestinal mast cell expansion and epithelial permeability are associated with reactivity to food proteins clinically, understanding the molecules that contribute towards developing anaphylaxis in sensitized individuals could guide the development of therapeutic interventions. By taking advantage of murine models susceptible to oral challenge, we characterized the developmental trajectory of intestinal mast cells and linked their tissue-intrinsic gene expression profiles with altered anaphylaxis requirements based on the route of exposure.

A speculative model to explain the requirements of mucosal mast cell-derived CysLTs in oral anaphylaxis could relate to rapid local activation of tissue ILC2s. In experimental food allergy, the function of ILC2s is IgE-dependent, and all three CysLTRs converge on ILC2 activation either through direct means or through the production of epithelial cues such as IL33 and IL25 and neuropeptides such as NMU (27–29, 33, 67, 68). This may explain why aLOX5 antagonism was capable of acutely preventing oral anaphylaxis, but blocking CysLTR1 or CysLTR2 individually was not. ILC2 activation triggers production of IL9 and IL13, augmenting intestinal mastocytosis and intestinal permeability respectively through secretory cell-associated antigen passages (SAPs) and paracellular pathways (9, 12, 14, 30). That histaminergic requirements for oral anaphylaxis were only observed in the most severely affected allergic mice agrees with leukotrienes representing a response capable of bridging ingestion to systemic exposure (64). Despite this, acute aLOX5 inhibition did not inhibit the passage of fluorescently labelled dextrans from intestine to blood nor inhibit systemic mast cell activation, suggesting a means by which leukotrienes affect readouts of anaphylaxis that are due to intestine-intrinsic actions such as activation of thermoregulatory neurons in the hindbrain (69).

The role of cysteinyl leukotrienes in human food allergy is poorly understood. While leukotrienes are detectible in the serum of patients undergoing anaphylaxis, it is not certain whether their inhibition can reduce reactions to oral allergen challenge (70, 71). In a retrospective analysis of children with food allergies, treatment with the CysLTR1 antagonist, pranlukast, reduced blood eosinophil counts, serum IgE, and serum IL4 and IL5 but was not associated with reduced severity of clinical reactions (72). On the other hand, a small retrospective study using montelukast sodium pretreatment acutely during oral immunotherapy (OIT), whereby individuals are given small doses of food antigens as a means of desensitization, reduced symptoms and helped patients achieve their target dose (73). Oral immunotherapy for food allergies itself appears to decrease the synthesis of eicosanoids including leukotrienes over the treatment course and reduces clinical reactivity (74). Whether mechanisms known to induce leukotriene production and reduce reactivity thresholds to food allergen ingestion, such as alcohol consumption, NSAID ingestion, or exercise (75–80) do so in leukotriene-dependent manners is another open question.

While the role of IgE in food allergic responses in humans is well accepted, the role of mucosal mast cells remains controversial (81). Local mast cell expansion has been observed histologically and functional differences described historically, but transcriptional profiling of mast cells from the intestinal mucosa of food-allergic individuals remains elusive (82–84). Most isolation protocols solely examine the lamina propria, which our data suggests may preclude analysis of mast cells within the epithelial layer. The developmental trajectory of intestinal mast cells we observed in food allergic mice was similar to that identified in nasal polyps of patients with Aspirin-Exacerbated Respiratory Disease (AERD), a population associated with leukotriene excess and atopy in humans (85). TGFb was associated with the transcriptional shaping of these intraepithelial mast cells, resulting in increased cysteinyl leukotriene secretion in humans. We propose that there may be common differentiation trajectories for mast cells at different mucosal barrier sites that are conserved across species. The scRNAseq data presented here may help to develop genetic tools enabling specific manipulation of mucosal mast cells to determine their roles in the body.

A hinderance towards the study of food allergies relates to differences in susceptibility of mouse background strains, with the widely used C57BL/6J being resistant to oral anaphylaxis. *Hoyt et al* report that a mutation affecting dipeptidase 1 (DPEP1), an epithelial brush-border enzyme that converts LTD4 to LTE4, underlies the susceptibility of the food allergy-prone C3H/HeJ mouse strain (86). Combined with the results presented here, these findings suggest that DPEP1 dysfunction may act to enhance the effects of intestinal mast cell degranulation at the epithelial interface. Together, these findings reveal a critical role of leukotrienes in anaphylactic reaction to ingested allergens.

## Materials and Methods

### Animals

All animal care and experimentation were approved by the Institutional Animal Care and Use Committee of Yale University School of Medicine and consistent with the National Institutes of Health, USA, guidelines. Mouse lines were interbred in our facilities to obtain the final strains described in the text. Genotyping was performed according to the protocols established for the respective strains by The Jackson Laboratories or published by the donating investigators. Mice were maintained at the Yale University animal facilities in temperature-(22°C) and humidity-controlled rooms, in a 12 h light/dark cycle with free access to standard chow diet (Teklad 2018S, Envigo) and water. Mice were euthanized by CO2 asphyxiation.

Female mice at 6–10 weeks of age were used for all experiments. BALB/cJ (000651), C57BL/6J(000664), C57BL/6 FcεRI KO (B6.129S2(Cg)-Fcer1atm1Knt/J, 010512), BALB/c Il4ra KO (BALB/c-Il4ratm1Sz/J, 003514)(87), aLOX5 KO (B6.129S2-Alox5^tm1Fun^/J)(88), LTC4sKO (C.129S7(B6)-Ltc4stm1Blam/J, 0309539)(21), CysLTR1 KO (C.B6-Cysltr1tm1Ykn/J, 030960)(26), CysLTR2 KO (C.B6-Cysltr2tm1Ykn/J,031718)(25) mice were purchased from The Jackson Laboratories and maintained in our facilities. BALB/cJ IgE KO mice were generously provided by H. C. Oettgen (Harvard University), and RMB mice (B6. Ms4a2tm1Mal310)(39) were generously provided by P. Launay (Université Paris Diderot). RMB mice, FcεRI KO, and IgE KO mice were backcrossed more than eight times onto BALB/cJ for this study. We used littermate controls in all experiments.

Chimaeric mice were generated as previously described (1). BALB/cJ wild-type or LTC4S-KO mice were used as donors in bone marrow transplant experiments. FceR1-DTR-TdTom RMB mice underwent a lethal total-body irradiation with two doses of 500 rad (Gammacell 40 ^137^Cs γ-irradiation source), with an interval of 3 h between the first and the second irradiation. Fresh, unseparated bone marrow cells (10 × 10^6^ per mouse) were injected into the tail vein of the irradiated recipient mice 4 h after lethal irradiation. Chimaerism efficiency was checked by flow cytometry 8 weeks post-irradiation and transplant using peripheral blood, and reconstituted mice were used 2 months after bone marrow transplantation.

### Drug Treatments

Zileuton 50mg/kg (Tocris), Montelukast Sodium 10mg/kg (Cayman Chemical Co) and HAMI3379 0.4mg/kg (Cayman Chemical Co), were administered 1 hour prior to each oral challenge, or starting 1 hour before the 5^th^ oral challenge as described in the text. Zileuton was administered via oral gavage in 0.5% methylcellulose, while Montelukast and HAMI3379 were delivered by intraperitoneal challenge in sterile PBS. All control groups received appropriate vehicle solutions(1). H1R inhibition in active and passive anaphylaxis experiments was achieved by treatment with 200 micrograms of Triprolidine 30 minutes prior to oral or systemic allergen challenge (61). The avb6 neutralizing monoclonal antibody 3G9 was generated as previously described (56). Mice were treated twice per week with 10 mg/kg of 3G9 or IgG1 isotype control (InVivoMAb) in InVivoPure pH 7.0 Dilution Buffer as previously described (89) with the first dose being given the morning prior to first oral challenge and last given within 4 days of takedown.

### Mast Cell Depletion

For mast cell depletion, sensitized RMB mice were injected i.p. with 0.05 mg/kg of diphtheria toxin (Sigma Aldrich D0564) three times every other day as previously described starting on day 14 (one week after the second s.c. sensitization)(1). Because this protocol was efficient at depleting mast cells even in heterozygous RMB mice, we used both mutants and heterozygotes as mast cell-depleted models.

### Experimental Food Allergy Model

The active food-allergy model using ovalbumin (OVA) and the adjuvant, aluminum hydrogel (Alum), has been previously described (1). Briefly, mice were sensitized on day 0 and day 7 by subcutaneous injection of 0.25mg/kg endotoxin-free ovalbumin (BIOVendor 321001) adsorbed in 50mg/kg aluminum hydroxide gel (alum, Invivogen vac-alu-250) and diluted to a final volume of 0.2mL in sterile phosphate buffered saline (PBS) pH=7.4. Controls received an antigen-free sensitization (referred to as PBS/alum or PBS). Mice were bled on day 14 for antibody titre measurements and subsequently challenged 7 times by oral gavages with 50 mg grade III OVA (Sigma-Aldrich A5378) in 0.2 mL of PBS on days 14, 16, 18, 20, 22, 24, and 26. In most experiments, mice were bled 1 hour after every other challenge for MCPT1 quantification as described below. Allergen-induced gastrointestinal transit time (GITT) measurements were performed on challenge 5, oral anaphylactic temperature drop on challenge 6, and the mice were humanely euthanized by CO2 asphyxiation for terminal analysis 1 hour after challenge 7.

The oral anaphylaxis paradigm using peanut/cholera toxin model was performed as previously described (35) except that the systemic peanut challenge was administered the week after oral challenge to confirm successful sensitization.

### Total Gastrointestinal Transit Time

Mice were sensitized and challenged as described in the “Experimental Food Allergy Model” section. On the 5^th^ oral challenge, mice were singly housed in cages with free access to food and water and were subsequently gavaged with 50mg of OVA mixed with 6% carmine red (C1022, Sigma-Aldrich) in 0.5% methylcellulose (M0512, Sigma-Aldrich). Mice were monitored every 15 minutes for the occurrence of diarrhea and/or red in the stool. GI transit time was measured as the time from oral gavage until carmine red dye was seen in the stool. Mice were co-house after the assay was complete for each mouse. In cases where Zileuton, Montelukast, HAMI3379, or drug cocktails were administered, mice were administered these reagents via the denoted routes and concentrations 1 hour prior to the 5^th^ oral challenge. Allergen-inducible GITT was represented as a fraction of each sensitized mouse’s GITT divided by the average of the GITT of un-sensitized mice within each group.

### Active Oral Anaphylaxis

Mice were sensitized and challenged as described in the “Experimental Food Allergy Model” section. After the 6^th^ oral gavage, the rectal temperature of each mouse was measured every 10 minutes for 1 hour via rectal thermometry (Thermalert TH5). In cases where mice were administered Zileuton, Montelukast, or HAMI3379, mice were administered these reagents via the denoted routes 1 hour prior to the 6^th^ oral challenge (as described above).

### Active Systemic Anaphylaxis Model

Mice were sensitized on day 0 and 7 with OVA adsorbed in Alum hydrogel as described above. On day 14, mice were bled for antibody levels to ensure proper sensitization. On day 16, these mice were challenged with 100 micrograms of grade V OVA intravenously. Temperature was measured every 15 minutes thereafter by rectal thermometry (Thermalert TH5). The greatest temperature drop was calculated by subtracting the baseline temperature of each mouse from the lowest temperature they reached during the recording time, with hematocrit being collected at 1 hour after challenge. Symptom scoring was performed as previously described (8) where 0 denoted no symptoms, 1=mouth/ear scratching with hind leg, 2=decreased activity, isolation, and puffiness around eyes/mouth, 3=motionless for >1min, lying prone on stomach, 4=no response to prodding or whisker stimuli, and 5=tremor, convulsion, and death.

### Generation of TNP-OVA

TNP-conjugated ovalbumin was generated as previously described with minor modifications (66). Briefly, 500mg of grade III OVA (Sigma) was resuspended in 10mL of 0.1M NaHCO3 buffer pH9.6 and mixed with 4mL of 25mg/mL TNP-e-Aminocaprolyl-OSU (ChemCruz, resuspended in DMSO). The resulting solution was wrapped in foil and mixed overnight on a rotating mixer. The following day, the TNP-OVA was purified using Amicon Ultra 4 3K Filter Devices (Sigma) and subsequently washed in-column 4 times with 4mL of sterile PBS. The TNP-OVA was then isolated from the column filter and resuspended in 2.5mL to yield a concentration of approximately 200mg/mL TNP-OVA. Efficiency of isolation of this approach was confirmed using Bradford assay and via a protein gel followed by Coomassie stain with an OVA standard.

### Passive IgE-Dependent Anaphylaxis Models

6–10 week old mice were primed on d0 with 10 micrograms of aDNP-IgE (Sigma, SPE7) intraperitoneally in 200 microliters of PBS and challenged intravenously 16–24 hrs later with 250 micrograms of DNP-HSA (LGC Bio Tech) in 100 microL of PBS. Temperature was measured prior to i.v. challenge and every 10 minutes afterwards for 1 hour. At 1 hr, mice were bled using hematocrit (Hct) tubes (BD Clay Adams Sure Prep Tubes) to measure vascular leak, and at 2 hours for MCPT1 measurements by ELISA. One week later, the same mice were administered 10 micrograms of aTNP-IgE (BD557080) in 200 microliters of PBS intraperitoneally. 16–24 hours later, they were challenged with 50mg of TNP-OVA (homemade, as described above) intragastrically in 250 microL of PBS. Body temperature was measured prior to gavage, and every 10 minutes after for 1 hour. Mice were bled at 1hr for Hct determination and 2 hours for MCPT1, as described above. In experiments pertaining to leukotriene deficiency’s effect on passive anaphylaxis, one week following passive oral anaphylaxis challenge, the small intestine was harvested to measure epithelial and lamina propria mast cells as described below, and serum was taken for MCPT1 measurements in the absence of allergen challenge.

### Toluidine Blue Mast Cell Degranulation Assay

Mast cell degranulation assessment was based on 1 micrometer Toluidine Blue-stained sections of ear pinna, bronchi, or stomach using a modified version of the protocol used in prior studies (43, 90). One hour after intragastric challenge with 50mg of OVA in sensitized or control mice treated with vehicle or 50mg/kg of Zileuton, or one hour after intravenous challenge with or without 250 micrograms of DNP-HSA in aDNP-IgE primed mice, mice were humanely euthanized with a lethal dose of ketamine/xylazine and fixed with a 10mL perfusion of ice cold PBS followed by 10 mL of 10% NBF. Tissues were then harvested and further fixed overnight in 10% NBF at room temperature. Single sections from each tissue were obtained, stained, and then submitted for slide-scanning at 40X resolution. Tissues were then annotated, mast cells counted in QuPath and classified as normal or degranulated based on 1) extent of granules exhibiting fusion with the cell membrane 2) granules identified immediately proximally to the cell of interest, and/or 3) alterations in staining/extrusion of the cell.

### Gastrointestinal Permeability Determination

Gastrointestinal permeability was measured via multiplexed intragastric gavage of 4kDa FITC-Dextran (20mg, Sigma Aldrich), and 70kDa TMR-Dextran (10mg, Sigma Aldrich) in 250 microliters of water per mouse as previously described (91). Briefly, mice 6–10 weeks old were fasted for 4 hours, treated with 50mg/kg of Zileuton or vehicle for 1 hour, gavaged with the above dextran mixture and serum was isolated 3 hrs later for determination of 4/70-kDa dextran by fluorescence using a SpectraMax M5 (Molecular Devices). Each mouse’s intestinal permeability was measured on d28 after 5 intragastric challenges with 50mg grade III OVA/mouse. For the last measurement (6^th^ oral challenge), 50mg OVA was mixed with the above tracers and gavaged together with serum harvested 3 hours later.

### Epithelial cell and lamina propria isolation

Single-cell suspensions of small intestinal epithelium and lamina propria were prepared as described (92). Briefly, the small intestine was isolated and opened longitudinally. Its contents were then rinsed in PBS following the removal of Peyer’s patches. The tissue was then cut into 2–3 cm segments and incubated in RPMI media (ThermoFisher) containing 5 mM EDTA, 0.145 mg/mL DTT, and 3% FBS at 37°C with 5% CO_2_ for 20 min with agitation. Pieces of intestine were washed in RPMI containing 2 mM EDTA to separate the epithelial fraction. Lamina propria digestion was performed using 0.1 mg/mL Liberase TL (Roche #540102001) and 0.5 mg/mL DNAse (DN25, Sigma-Aldrich) in RPMI for 30 min at 37°C with 5% CO_2_. Digested tissue was sequentially strained through 70 micron and 40 micron strainers and washed in RPMI containing 3% FBS. The epithelial fraction was also subjected to a 30% Percoll (GE17–0891-01, Sigma Aldrich) density gradient by centrifugation in preparation for sorting. Cells were then stained for flow cytometric analysis.

### Small Intestinal Mast Cell Flow Cytometry and FACS

Single-cell suspensions were treated with anti-CD16/32 (Fc block) (14–9161-73, ThermoFisher) and stained with either ethidium monoazide bromide (ThermoFisher #E1374) in 2% FBS in PBS or Zombie Yellow (Biolegend #423103) in PBS to identify live and dead cells. Cells were then stained with a selection of the following antibodies at a concentration of 1 mg/mL except where otherwise indicated: FITC-IgE (clone R35–72; BD Biosciences #553415), PE-FceRI (clone MAR-1; eBioscience 12–5898-82), PE-integrin beta7 (clone FIB504; eBioscience 12–5867-42), APC-CD11b (clone M1/70; ThermoFisher #17–0112-82), APC-CD8a (clone 53–6.7; BD Biosciences #553035), PE/Cy7-CD117 (clone 2B8; eBioscience #25–1171-82), APC/eFluor780-CD19 (clone eBio1D3; eBioscience #47–0193-82), Alexa700-CD3 (clone 17A2; Biolegend #100216), Alexa700-CD4 (clone RM4–5; Invitrogen #56–0042-82), BUV395-CD45 (clone 30-F11; BD Biosciences #564279), eFluor450-FceRI (clone MAR-1; eBioscience 48–5898-82), PerCP-eFluor710-CD49d (Integrin alpha 4) (clone R1–2; eBioscience 46–0492-82), BUV737-CD103 (Integrin alpha E) (clone 2E7; eBioscience #367–1031-82), biotin-ST2 (clone DJ8; MDBioproducts #101001B), and SuperBright600-Streptavidin (Invitrogen #63–4317-82). Cells were fixed with 1.6% paraformaldehyde (Electron Microscopy Sciences, #15710). Flow cytometry was performed using either a BD LSRII analyser equipped with the following lasers: 355 nm (UV), 405 nm (violet), 488 nm (blue), and 633 nm (red) or a BD Symphony analyzer equipped with the following lasers: 355nm (UV), 405nm (violet), 488nm (blue), 561nm (yellow green), 637nm (red), and 780nm (NIR). Data was analysed using FlowJo software. Gates were drawn according to fluorescence minus one (FMO) controls.

### Histological Quantification of Intestinal Mast Cell

The middle 10cm of small intestine was harvested 1 hour after the 7^th^ OVA gavage, flushed once intraluminally with 1xPBS, followed by once with 10% NBF. Intestines were then opened longitudinally, and fixed overnight on Whatman paper in 10% NBF at room temperature and subsequently rolled into swiss rolls as previously described. The intestines were then dehydrated, embedded in paraffin, cut, and stained via naphthol chloroacetate-esterase stain by the Yale Pathology Tissue Service. Mast cells were quantified by counting 5 independent 10x FOV for CAE+ cells and averaging them to obtain an average per independent sample.

### Total and OVA-Specific Antibody Quantification

Serum levels of total IgE and OVA-specific IgE and IgG1 antibodies were determined by ELISA, as previously described (1), with slight modifications for OVA-specific IgE. For total IgE, ELISA-grade plates (490012–252, VWR) were coated overnight at 4°C with 2 mg/mL of anti-mouse IgE (553413, clone R35–72, BD Pharmingen) in 0.1 M sodium carbonate buffer pH 9.5. Plates were blocked with 1% BSA (Fisher BP1600–1) for 2 h at room temperature. Serum from sensitized and control mice was diluted up to 1:50 and incubated for 2 h at room temperature. Purified mouse IgE (BD Biosciences 557079) was used as the standard with the highest concentration of 100 ng/mL followed by two-fold dilutions to create a standard curve. Afterwards, 500 ng/mL of biotin-conjugated anti-IgE detection antibodies (553419, clone R35–118, BD Biosciences) were incubated for 1 h at room temperature followed with another incubation of diluted HRP-conjugated streptavidin (554066, BD Biosciences) for 30 min at room temperature. Plates were then incubated in the dark at room temperature with TMB substrate reagent (555214, BD Biosciences) until developed. Plates were read at 450 nm immediately after adding stop solution (3M H_2_SO_4_). Between each step, plates were washed five times with 0.05% Tween-20 in PBS. OVA-specific IgG1 antibodies in the serum were assayed by direct ELISA. Plates were coated overnight at 4°C with 20 mg/mL of grade V OVA (A5503, Sigma-Aldrich) in coating buffer (0.1 M sodium carbonate pH 9.5). After the blocking step, serum samples were diluted up to 1:10,000 and incubated for 1 hr at room temperature. Purified mouse BALB/c IgG1 (557273, clone MOPC-31C, BD Biosciences) was used as the standard with the highest concentration at 100 ng/mL. Biotin-conjugated mouse IgG1 (553441, clone A85–1, BD Biosciences) was used for detection at 100 ng/mL. The HRP-conjugated streptavidin, substrate reagents, blocking and washing solutions used for the IgG1 ELISA were the same as described above for IgE ELISAs. Serum concentrations of OVA-specific IgE were also assayed by direct ELISA method. Plates were coated overnight at 4°C with 100 microg/mL of grade V OVA (A5503, Sigma-Aldrich) in coating buffer (0.1 M sodium carbonate pH 9.5). After the blocking step, serum samples were diluted up to 1:50 and incubated for 1 hr at room temperature. Purified mouse anti-OVA IgE (7091, clone E-C1, Chondrex) was used as the standard with the highest concentration at 3 microg/mL Bound OVA-specific IgE was detected by incubation with a mouse IgE-HRP (Southern Biotech, 1110–05) for 1 hour at RT. Development using TMB, quenching with H2SO4, and detection was performed as above.

### MCPT1 Quantification

Serum was collected at either day 14 (after sensitization but prior to oral challenge) or 1 hour after 50mg OVA (III) i.g. administration on challenges 2, 4, and 7. MCPT1 was quantified by ELISA (Invitrogen, Cat#88–7503-88). For passive anaphylaxis experiments, serum MCPT1 measurements were made at 2hr post challenge instead.

### *In vitro* Mucosal Mast Cell Imprinting by TGFb and mediator quantification

Bone marrow was isolated from the femurs and tibia of adult female BALB/cJ mice and passed through a 70 micron strainer to produce a single cell suspension. Isolated bone marrow cells were cultured in DMEM supplemented with 25 mM HEPES, 1% pen/strep, 1% non-essential amino acids, 2 mM L-glutamine, 1% sodium pyruvate, 10% fetal bovine serum, 50 uM β-mercaptoethanol, and 30 ng/mL recombinant mouse IL-3 (Peprotech, 213–13) for at least 6 weeks to generate bone marrow-derived mast cells. Mast cell purity was observed to be at least 95% based on expression of Fcer1a and c-Kit. These bone marrow mast cells were then cultured with 5 ng/mL IL-3 (Peprotech, 213–13) and 100 ng/mL SCF (Peprotech, 250–03), with or without 25 ng/mL TGF-B1(R&D, 240-B-003) for 7 days as previously described (52). On the 7^th^ day, the mast cells were collected for RNA isolation or flow cytometric analysis, described below. In experiments involving antigen stimulation, mast cells were sensitized with 2 ug/mL anti-DNP IgE (Sigma, SPE7) on the 6^th^ day. On the 7^th^ day, they were washed 3 times with serum-free media and then challenged with 10 ng/mL DNP-HSA (LGC Bio Tech) in serum-free media for 30 minutes. Afterwards, the media was collected for ELISA for MCPT1 (as previously described), PGD2 (Cayman Chemical, 512031), CysLTs (Cayman Chemical, 500390), and Histamine (Cayman Chemical, 589651) quantification.

### Flow cytometric evaluation of *in vitro* bone marrow mast cell cultures

Bone marrow mast cells were first incubated with Zombie Yellow (1:500 in PBS) for 15 minutes at room temperature. Then they were washed and incubated with anti-CD16/32 (Fc block, 1:400), anti-CD117-PE-Cy7 (1:400), anti-Fcer1a-FITC (1:200), anti-LPAM1-BV421 (1:100), anti-CD103-BV711 (1:100), and anti-ST2-PE (1:80), or the corresponding isotype controls in PBS + 0.5% BSA for 20 minutes on ice. For detection of MCPT1, mMCPT1 antibody (TG6.1, Invitrogen), was conjugated to Alexa Flour 647 using AF647 Labelling Kit (Invitrogen) according to the manufacturer’s instructions. For MCPT-1 staining, the cells were then fixed and permeabilized with the BD Cytofix/Cytoperm kit according to the manufacturer’s instructions, followed by incubation with anti-MCPT1-AF647 (1:200) in permeabilization buffer for 20 minutes on ice. 123count eBeads (Thermo Fisher) were added to the samples for cell number quantitation. Flow cytometry was conducted with a BD FACSymphony Cell Analyzer and the data were analyzed with FlowJo.

### RNA Isolation, cDNA synthesis, and qRT-PCR

RNA was isolated from samples using an RNeasy Micro Kit (Qiagen) and cDNA was synthesized using MMLV reverse transcriptase (Clontech) with oligo(dT) primers. qRT PCR was performed on CFX384 Real-Time System (Bio-Rad) using PerfeCTa SYBR Green Supermix (Quanta Biosciences) and gene-specific primers, and transcripts were normalized to Rpl13a. A list of the primers utilized in this study can be found in Table S7.

### Bulk RNA Sequencing

RNA was extracted from sorted mast cells using an RNeasy Plus Micro Kit (Qiagen), and library construction was performed using a SMART-seq2 protocol (93). Sequencing libraries were sequenced on an Illumina NextSeq 500 (38×38 bp paired-end run). Sequencing reads were aligned to the mouse transcriptome (GRCm38 ensembl v101; cDNA and ncRNA) and quantified by Kallisto (v0.45.0)(94) with a k-mer index 25 and 60 bootstrapping. The expression of transcript was calculated in TPM (transcripts per million). The expression of the gene was calculated by aggregating the TPM of all matched transcripts. Statistical analyses for differentially expressed genes were performed by tximport and DESeq2 (95).

### Intestinal mast cell single cell analysis

Mice were sensitized against OVA and challenged intragastrically five times with OVA as described above. Small intestinal lamina propria and epithelial fractions of immune cells were subsequently isolated and mast cells from each fraction sorted using a FACS Aria cell sorter into ice cold 10% FBS in dPBS w/o Ca, Mg, or EDTA. Sorted cells from two sensitized mice (two LP and two IEL samples) were then spun down, counted, and prepared separately using the Chromium GEM-X Single Cell 3’ v4 assay (10X Genomics) according to the manufacturer’s protocol at the Keck Microarray Shared Resource at Yale School of Medicine. Libraries were sequenced on a NovaSeq 6000 by the Yale Center for Genome Analysis.

### Quality control and single cell RNA sequencing (scRNAseq) analysis

The data quality control and processing were performed with Seurat (96) and R packages (R version 4.3.3). 31934 cells from all samples were merged initially. For quality control, we used quality metrics for the initial filtering. Cell complexity score was calculated by using the formula $Log10(\frac{Number\;of\;genes}{Number\;of\;UMI})$ (UMI = Unique Molecular Identifier) as an index to measure the potential artifact or contamination in the samples. Cells with > 500 UMI count, > 500 genes, and > 0.8 complexity score were retained. After exclusion of poor-quality cells, we were left with 23333 cells. We processed the data by running Seurat v5 pipeline. The raw count of the retained data was normalized, and variable features were identified with NormalizeData and FindVariableFeatures functions, respectively. We next linearly transformed the normalized data using the ScaleData function. RunPCA and RunUMAP were performed for dimension reduction. All data samples were integrated using the Harmony package (51). After integration, 14 clusters were identified using FindNeighbors and FindClusters on integrated data with a resolution of 0.6. Of these, we noted that clusters 8, 9, 11, 12, 13 expressed markers prototypical for B cells, smooth muscle cells, and epithelial cell types (differentially expressed marker genes such as Ighm, Myocd, Vwf, Cnn1, etc.). Thus, for analysis of mast cell intrinsic transcripts, we excluded cells on the basis of B cells, smooth muscle cells, and epithelial cells, which left us with 22171 number of intestinal mast cells expressing *FcεRI* and c-*Kit* markers. The intestinal mast cells were then re-clustered into 9 identified clusters. We next assessed the differentially expressed genes (DEGs) using the FindMarkers function with a) an adjusted p value <0.05; b) an average Log2 fold change > 1 or < −1; c) a percentage of cells (pct) where the feature was detected > 0.1 in either group. The potential differentiation trajectory was inferred and plotted using Monocle3 toolkits (97). Differentially expressed genes between clusters 0,1 versus cluster 2 with the absolute log2 fold change >= 1 and the adjusted p value < 0.05 were subset for the downstream Ingenuity Pathway Analysis (IPA) and Gene ontology (GO) enrichment analysis. For IPA analysis upstream regulators of the differentially expressed genes between cluster 0,1 versus cluster 2 were predicted and interpreted using the Core Analysis function in the IPA software (QIAGEN; version 01–23-01). GO enrichment of the differentially expressed genes between cluster 0,1 versus cluster 2 (with average Log2 fold change >1 or <−1 and adjusted p value <0.05) was performed using the clusterProfiler R package (98) based on the hallmark gene set from the Molecular Signatures Database (MSigDB)(99), with pathway p value cutoff of 0.5 and q value cutoff of 1 after p value adjustment using Benjamini–Hochberg (BH) multiple testing. An additional dataset of mast cells from the gut, peritoneal cavity, and skin was downloaded from Tauber et al (59) and processed as their descriptions All plots in this analysis were generated by the Seurat visualization methods, the ggplot2, and the EnhancedVolcano packages.

### Statistical analysis:

All statistical analyses except for Bulk RNAseq data were performed using Graphpad Prism Software. Exact tests utilized are denoted in figure legend or in supplemental text. Statistical significance is defined as *p<0.05, **p<0.01, ***p<0.001, and ****p<0.0001.

## Supplementary Material

Table S1

Table S2

Table S3

Table S4

Table S5

Table S6

Table S7

Supplementary Materials

Supplementary Materials

Figs. S1–S9

Tables S1–S7

## Data Availability

All data needed to evaluate the conclusions in the paper are present in the paper or the Supplementary Materials. Bulk sequencing and scRNA sequencing of intestinal mast cells generated for this paper can be accessed through the Gene Expression Ombibus under accession no. GSE293906. Code utilized in the analysis of this data to produce each figure can be found on GitHub (100). Single cell RNAseq of mast cells in different tissues used to generate Fig 3a can be found at the non-profit data repository Dryad (59).
