# Supplementary material for: Intestinal mast cell-derived leukotrienes mediate anaphylactic response to ingested antigens
Source: Science. Author manuscript; Available in PMC 2025 Oct 10. (PMC12513082; doi:10.1126/science.adp0246)
Supplement: Supplementary Materials [file NIHMS2111023-supplement-Supplementary_Materials.docx]

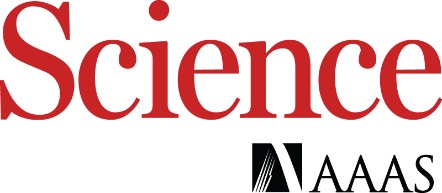


Supplementary Materials for

**Intestinal mast cell-derived leukotrienes mediate anaphylactic response to ingested antigens**

Nathaniel D. Bachtel^1^, Jaime L. Cullen^1^, Min Liu^1^, Steven A. Erickson^1^, Vassily I. Kutyavin^1^, Darine W. El-Naccache^1^, Esther B. Florsheim^2^, Jaechul Lim^3^, Zuri A. Sullivan^4^, Raiden Imaeda^1^, Andrew Hudak^1^, Cuiling Zhang^1^, Ruslan Medzhitov^1,5,6^ *

**Affiliations:**

^1^ Department of Immunobiology, Yale University School of Medicine, New Haven, Connecticut 06520, USA.

^2^School of Life Sciences, Arizona State University, Tempe, Arizona 85287-7501, USA.

^3^College of Veterinary Medicine and Research Institute for Veterinary Science, Seoul National University, Seoul 08826, Republic of Korea.

^4^Department of Molecular and Cellular Biology, Harvard University, Cambridge, MA

^5^Howard Hughes Medical Institute.

^6^Tananbaum Center for Theoretical and Analytical Human Biology, Yale University School of Medicine, New Haven, CT, USA

*Corresponding author. Email: [ruslan.medzhitov@yale.edu](mailto:ruslan.medzhitov@yale.edu)

**The PDF file includes:**

Figs. S1 to S9

Tables S1-7

References (1-100)

References

1. E. B. Florsheim *et al.*, Immune sensing of food allergens promotes avoidance behaviour. *Nature* **620**, 643-+ (2023).

2. E. B. Florsheim, Z. A. Sullivan, W. Khoury-Hanold, R. Medzhitov, Food allergy as a biological food quality control system. *Cell* **184**, 1440-1454 (2021).

3. M. Profet, The function of allergy: immunological defense against toxins. *Q Rev Biol* **66**, 23-62 (1991).

4. T. Plum *et al.*, Mast cells link immune sensing to antigen-avoidance behaviour. *Nature* **620**, 634-642 (2023).

5. N. W. Palm, R. K. Rosenstein, R. Medzhitov, Allergic host defences. *Nature* **484**, 465-472 (2012).

6. S. Anvari, J. Miller, C. Y. Yeh, C. M. Davis, IgE-Mediated Food Allergy. *Clin Rev Allergy Immunol* **57**, 244-260 (2019).

7. F. D. Finkelman, Anaphylaxis: lessons from mouse models. *J Allergy Clin Immunol* **120**, 506-515; quiz 516-507 (2007).

8. K. Arias *et al.*, Concurrent blockade of platelet-activating factor and histamine prevents life-threatening peanut-induced anaphylactic reactions. *J Allergy Clin Immunol* **124**, 307-314, 314 e301-302 (2009).

9. T. K. Noah *et al.*, IL-13-induced intestinal secretory epithelial cell antigen passages are required for IgE-mediated food-induced anaphylaxis. *J Allergy Clin Immunol* **144**, 1058-1073 e1053 (2019).

10. R. Ahrens *et al.*, Intestinal mast cell levels control severity of oral antigen-induced anaphylaxis in mice. *Am J Pathol* **180**, 1535-1546 (2012).

11. E. B. Brandt *et al.*, Mast cells are required for experimental oral allergen-induced diarrhea. *J Clin Invest* **112**, 1666-1677 (2003).

12. C. Y. Chen *et al.*, Induction of Interleukin-9-Producing Mucosal Mast Cells Promotes Susceptibility to IgE-Mediated Experimental Food Allergy. *Immunity* **43**, 788-802 (2015).

13. J. M. Leyva-Castillo *et al.*, Mechanical Skin Injury Promotes Food Anaphylaxis by Driving Intestinal Mast Cell Expansion. *Immunity* **50**, 1262-1275 e1264 (2019).

14. E. E. Forbes *et al.*, IL-9- and mast cell-mediated intestinal permeability predisposes to oral antigen hypersensitivity. *J Exp Med* **205**, 897-913 (2008).

15. H. Osterfeld *et al.*, Differential roles for the IL-9/IL-9 receptor α-chain pathway in systemic and oral antigen-induced anaphylaxis. *J Allergy Clin Immun* **125**, 469-476 (2010).

16. J. Z. Haeggstrom, C. D. Funk, Lipoxygenase and leukotriene pathways: biochemistry, biology, and roles in disease. *Chem Rev* **111**, 5866-5898 (2011).

17. J. D. Clark, N. Milona, J. L. Knopf, Purification of a 110-kilodalton cytosolic phospholipase A2 from the human monocytic cell line U937. *Proc Natl Acad Sci U S A* **87**, 7708-7712 (1990).

18. G. K. Reid *et al.*, Correlation between Expression of 5-Lipoxygenase-Activating Protein, 5-Lipoxygenase, and Cellular Leukotriene Synthesis. *J Biol Chem* **265**, 19818-19823 (1990).

19. B. Samuelsson, S. E. Dahlen, J. A. Lindgren, C. A. Rouzer, C. N. Serhan, Leukotrienes and lipoxins: structures, biosynthesis, and biological effects. *Science* **237**, 1171-1176 (1987).

20. B. K. Lam, J. F. Penrose, G. J. Freeman, K. F. Austen, Expression cloning of a cDNA for human leukotriene C4 synthase, an integral membrane protein conjugating reduced glutathione to leukotriene A4. *Proc Natl Acad Sci U S A* **91**, 7663-7667 (1994).

21. Y. Kanaoka, A. Maekawa, J. F. Penrose, K. F. Austen, B. K. Lam, Attenuated zymosan-induced peritoneal vascular permeability and IgE dependent passive cutaneous anaphylaxis in mice lacking leukotriene C4 synthase. *J Biol Chem* **276**, 22608-22613 (2001).

22. M. E. Anderson, R. D. Allison, A. Meister, Interconversion of leukotrienes catalyzed by purified gamma-glutamyl transpeptidase: concomitant formation of leukotriene D4 and gamma-glutamyl amino acids. *Proc Natl Acad Sci U S A* **79**, 1088-1091 (1982).

23. E. M. Kozak, S. S. Tate, Glutathione-degrading enzymes of microvillus membranes. *J Biol Chem* **257**, 6322-6327 (1982).

24. Y. Kanaoka, A. Maekawa, K. F. Austen, Identification of GPR99 Protein as a Potential Third Cysteinyl Leukotriene Receptor with a Preference for Leukotriene E4 Ligand. *J Biol Chem* **288**, 10967-10972 (2013).

25. T. C. Beller, A. Maekawa, D. S. Friend, K. F. Austen, Y. Kanaoka, Targeted gene disruption reveals the role of the cysteinyl leukotriene 2 receptor in increased vascular permeability and in bleomycin-induced pulmonary fibrosis in mice. *J Biol Chem* **279**, 46129-46134 (2004).

26. A. Maekawa, K. F. Austen, Y. Kanaoka, Targeted gene disruption reveals the role of cysteinyl leukotriene 1 receptor in the enhanced vascular permeability of mice undergoing acute inflammatory responses. *J Biol Chem* **277**, 20820-20824 (2002).

27. T. Liu *et al.*, Type 2 Cysteinyl Leukotriene Receptors Drive IL-33-Dependent Type 2 Immunopathology and Aspirin Sensitivity. *J Immunol* **200**, 915-927 (2018).

28. J. von Moltke *et al.*, Leukotrienes provide an NFAT-dependent signal that synergizes with IL-33 to activate ILC2s. *J Exp Med* **214**, 27-37 (2017).

29. L. G. Bankova *et al.*, The cysteinyl leukotriene 3 receptor regulates expansion of IL-25-producing airway brush cells leading to type 2 inflammation. *Sci Immunol* **3**, (2018).

30. J. B. Lee *et al.*, IL-25 and CD4 T cells enhance type 2 innate lymphoid cell-derived IL-13 production, which promotes IgE-mediated experimental food allergy. *J Allergy Clin Immun* **137**, 1216-+ (2016).

31. H. Xu *et al.*, Transcriptional Atlas of Intestinal Immune Cells Reveals that Neuropeptide alpha-CGRP Modulates Group 2 Innate Lymphoid Cell Responses. *Immunity* **51**, 696-708 e699 (2019).

32. P. Licona-Limon, L. K. Kim, N. W. Palm, R. A. Flavell, TH2, allergy and group 2 innate lymphoid cells. *Nat Immunol* **14**, 536-542 (2013).

33. J. von Moltke, M. Ji, H. E. Liang, R. M. Locksley, Tuft-cell-derived IL-25 regulates an intestinal ILC2-epithelial response circuit. *Nature* **529**, 221-225 (2016).

34. L. M. Bartnikas *et al.*, Epicutaneous sensitization results in IgE-dependent intestinal mast cell expansion and food-induced anaphylaxis. *J Allergy Clin Immunol* **131**, 451-460 e451-456 (2013).

35. J. A. Gertie *et al.*, Oral anaphylaxis to peanut in a mouse model is associated with gut permeability but not with Tlr4 or Dock8 mutations. *J Allergy Clin Immun* **149**, 262-274 (2022).

36. K. Orgel *et al.*, Genetic diversity between mouse strains allows identification of the CC027/GeniUnc strain as an orally reactive model of peanut allergy. *J Allergy Clin Immunol* **143**, 1027-1037 e1027 (2019).

37. F. D. Finkelman *et al.*, IL-4 is required to generate and sustain in vivo IgE responses. *J Immunol* **141**, 2335-2341 (1988).

38. U. Blank *et al.*, Complete structure and expression in transfected cells of high affinity IgE receptor. *Nature* **337**, 187-189 (1989).

39. A. Dahdah *et al.*, Mast cells aggravate sepsis by inhibiting peritoneal macrophage phagocytosis. *J Clin Invest* **124**, 4577-4589 (2014).

40. K. Arias *et al.*, Distinct immune effector pathways contribute to the full expression of peanut-induced anaphylactic reactions in mice. *J Allergy Clin Immun* **127**, 1552-U1359 (2011).

41. F. Jonsson *et al.*, Mouse and human neutrophils induce anaphylaxis. *J Clin Invest* **121**, 1484-1496 (2011).

42. I. Miyajima *et al.*, Systemic anaphylaxis in the mouse can be mediated largely through IgG1 and Fc gammaRIII. Assessment of the cardiopulmonary changes, mast cell degranulation, and death associated with active or IgE- or IgG1-dependent passive anaphylaxis. *J Clin Invest* **99**, 901-914 (1997).

43. Y. Taketomi *et al.*, Lipid-orchestrated paracrine circuit coordinates mast cell maturation and anaphylaxis through functional interaction with fibroblasts. *Immunity* **57**, 1828-1847 e1811 (2024).

44. N. Nakano, J. Kitaura, Mucosal Mast Cells as Key Effector Cells in Food Allergies. *Cells-Basel* **11**, (2022).

45. J. Bienenstock, A. D. Befus, F. Pearce, J. Denburg, R. Goodacre, Mast cell heterogeneity: derivation and function, with emphasis on the intestine. *J Allergy Clin Immunol* **70**, 407-412 (1982).

46. D. F. Dwyer *et al.*, Human airway mast cells proliferate and acquire distinct inflammation-driven phenotypes during type 2 inflammation. *Sci Immunol* **6**, (2021).

47. B. D. McNeil *et al.*, Identification of a mast-cell-specific receptor crucial for pseudo-allergic drug reactions. *Nature* **519**, 237-241 (2015).

48. M. F. Gurish *et al.*, Intestinal mast cell progenitors require CD49dbeta7 (alpha4beta7 integrin) for tissue-specific homing. *J Exp Med* **194**, 1243-1252 (2001).

49. R. Gentek *et al.*, Hemogenic Endothelial Fate Mapping Reveals Dual Developmental Origin of Mast Cells. *Immunity* **48**, 1160-1171 e1165 (2018).

50. L. G. Bankova, D. F. Dwyer, A. Y. Liu, K. F. Austen, M. F. Gurish, Maturation of mast cell progenitors to mucosal mast cells during allergic pulmonary inflammation in mice. *Mucosal Immunol* **8**, 596-606 (2015).

51. I. Korsunsky *et al.*, Fast, sensitive and accurate integration of single-cell data with Harmony. *Nat Methods* **16**, 1289-1296 (2019).

52. T. Derakhshan *et al.*, Lineage-specific regulation of inducible and constitutive mast cells in allergic airway inflammation. *J Exp Med* **218**, (2021).

53. L. Yang *et al.*, Intraepithelial mast cells drive gasdermin C-mediated type 2 immunity. *Immunity* **57**, 1056-1070 e1055 (2024).

54. P. A. Knight *et al.*, Enteric expression of the integrin αvβ6 is essential for nematode-induced mucosal mast cell hyperplasia and expression of the granule chymase, mouse mast cell protease-1. *American Journal of Pathology* **161**, 771-779 (2002).

55. K. Sugimoto *et al.*, The alphavbeta6 integrin modulates airway hyperresponsiveness in mice by regulating intraepithelial mast cells. *J Clin Invest* **122**, 748-758 (2012).

56. P. H. Weinreb *et al.*, Function-blocking integrin alphavbeta6 monoclonal antibodies: distinct ligand-mimetic and nonligand-mimetic classes. *J Biol Chem* **279**, 17875-17887 (2004).

57. Y. Makabe-Kobayashi *et al.*, The control effect of histamine on body temperature and respiratory function in IgE-dependent systemic anaphylaxis. *J Allergy Clin Immunol* **110**, 298-303 (2002).

58. M. Tauber *et al.*, Landscape of mast cell populations across organs in mice and humans. *J Exp Med* **220**, (2023).

59. N. Gaudenzio *et al.*, Landscape of Mast cell populations across organs in mice and humans. *Dryad*, (2023).

60. J. B. Wechsler, H. A. Schroeder, A. J. Byrne, K. B. Chien, P. J. Bryce, Anaphylactic responses to histamine in mice utilize both histamine receptors 1 and 2. *Allergy* **68**, 1338-1340 (2013).

61. R. T. Strait, S. C. Morris, M. Y. Yang, X. W. Qu, F. D. Finkelman, Pathways of anaphylaxis in the mouse. *J Allergy Clin Immun* **109**, 658-668 (2002).

62. S. Ualiyeva *et al.*, Tuft cell-produced cysteinyl leukotrienes and IL-25 synergistically initiate lung type 2 inflammation. *Sci Immunol* **6**, eabj0474 (2021).

63. J. W. McGinty *et al.*, Tuft-Cell-Derived Leukotrienes Drive Rapid Anti-helminth Immunity in the Small Intestine but Are Dispensable for Anti-protist Immunity. *Immunity* **52**, 528-541 e527 (2020).

64. R. T. Strait *et al.*, Ingested allergens must be absorbed systemically to induce systemic anaphylaxis. *J Allergy Clin Immun* **127**, 982-U234 (2011).

65. Z. Y. Kucuk *et al.*, Induction and suppression of allergic diarrhea and systemic anaphylaxis in a murine model of food allergy. *J Allergy Clin Immunol* **129**, 1343-1348 (2012).

66. R. T. Strait, S. C. Morris, F. D. Finkelman, IgG-blocking antibodies inhibit IgE-mediated anaphylaxis in vivo through both antigen interception and FcγRIIb cross-linking. *J Clin Invest* **116**, 833-841 (2006).

67. O. T. Burton *et al.*, IgE promotes type 2 innate lymphoid cells in murine food allergy. *Clin Exp Allergy* **48**, 288-296 (2018).

68. C. S. N. Klose *et al.*, The neuropeptide neuromedin U stimulates innate lymphoid cells and type 2 inflammation. *Nature* **549**, 282-286 (2017).

69. C. Bao *et al.*, A mast cell-thermoregulatory neuron circuit axis regulates hypothermia in anaphylaxis. *Sci Immunol* **8**, eadc9417 (2023).

70. C. Denzlinger, C. Haberl, W. Wilmanns, Cysteinyl leukotriene production in anaphylactic reactions. *Int Arch Allergy Immunol* **108**, 158-164 (1995).

71. E. Ono *et al.*, Increased production of cysteinyl leukotrienes and prostaglandin D2 during human anaphylaxis. *Clin Exp Allergy* **39**, 72-80 (2009).

72. Y. Yamakawa *et al.*, Effects of leukotriene receptor antagonists on peripheral eosinophil counts and serum IgE levels in children with food allergy. *Drugs R D* **10**, 147-154 (2010).

73. M. Takahashi *et al.*, New efficacy of LTRAs (montelukast sodium): it possibly prevents food-induced abdominal symptoms during oral immunotherapy. *Allergy Asthma Clin Immunol* **10**, 3 (2014).

74. Y. V. Virkud *et al.*, Immunomodulatory metabolites in IgE-mediated food allergy and oral immunotherapy outcomes based on metabolomic profiling. *Pediatr Allergy Immunol* **35**, e14267 (2024).

75. F. Wolbing, J. Fischer, M. Koberle, S. Kaesler, T. Biedermann, About the role and underlying mechanisms of cofactors in anaphylaxis. *Allergy* **68**, 1085-1092 (2013).

76. I. Bjarnason *et al.*, Mechanisms of Damage to the Gastrointestinal Tract From Nonsteroidal Anti-Inflammatory Drugs. *Gastroenterology* **154**, 500-514 (2018).

77. J. Sanchez-Lopez *et al.*, Food-dependent NSAID-induced hypersensitivity (FDNIH) reactions: Unraveling the clinical features and risk factors. *Allergy* **76**, 1480-1492 (2021).

78. S. Dua *et al.*, Effect of sleep deprivation and exercise on reaction threshold in adults with peanut allergy: A randomized controlled study. *J Allergy Clin Immunol* **144**, 1584-1594 e1582 (2019).

79. P. J. Manning *et al.*, Inhibition of exercise-induced bronchoconstriction by MK-571, a potent leukotriene D4-receptor antagonist. *N Engl J Med* **323**, 1736-1739 (1990).

80. F. Bishehsari *et al.*, Alcohol and Gut-Derived Inflammation. *Alcohol Res* **38**, 163-171 (2017).

81. R. A. Wood *et al.*, Omalizumab for the Treatment of Multiple Food Allergies. *N Engl J Med* **390**, 889-899 (2024).

82. L. Enerback, Mucosal mast cells in the rat and in man. *Int Arch Allergy Appl Immunol* **82**, 249-255 (1987).

83. C. C. Fox, A. M. Dvorak, S. P. Peters, A. Kagey-Sobotka, L. M. Lichtenstein, Isolation and characterization of human intestinal mucosal mast cells. *J Immunol* **135**, 483-491 (1985).

84. F. L. Pearce *et al.*, Functional characteristics of mucosal and connective tissue mast cells of man, the rat and other animals. *Int Arch Allergy Appl Immunol* **77**, 274-276 (1985).

85. T. Derakhshan *et al.*, Human intraepithelial mast cell differentiation and effector function are directed by TGF-β signaling. *J Clin Invest* **135**, (2025).

86. L. R. Hoyt *et al.*, Cysteinyl leukotrienes stimulate gut absorption of food allergens and promote anaphylaxis in mice. *Science*, (2025).

87. N. NobenTrauth *et al.*, An interleukin 4 (IL-4)-independent pathway for CD4(+) T cell IL-4 production is revealed in IL-4 receptor-deficient mice. *P Natl Acad Sci USA* **94**, 10838-10843 (1997).

88. X. S. Chen, J. R. Sheller, E. N. Johnson, C. D. Funk, Role of Leukotrienes Revealed by Targeted Disruption of the 5-Lipoxygenase Gene. *Nature* **372**, 179-182 (1994).

89. Z. W. Peng *et al.*, Integrin alphavbeta6 critically regulates hepatic progenitor cell function and promotes ductular reaction, fibrosis, and tumorigenesis. *Hepatology* **63**, 217-232 (2016).

90. T. Takeishi, T. R. Martin, I. M. Katona, F. D. Finkelman, S. J. Galli, Differences in the expression of the cardiopulmonary alterations associated with anti-immunoglobulin E-induced or active anaphylaxis in mast cell-deficient and normal mice. Mast cells are not required for the cardiopulmonary changes associated with certain fatal anaphylactic responses. *J Clin Invest* **88**, 598-608 (1991).

91. S. D. Chanez-Paredes, S. Abtahi, W. T. Kuo, J. R. Turner, Differentiating Between Tight Junction-Dependent and Tight Junction-Independent Intestinal Barrier Loss In Vivo. *Methods Mol Biol* **2367**, 249-271 (2021).

92. Z. A. Sullivan *et al.*, γδ T cells regulate the intestinal response to nutrient sensing. *Science* **371**, 1223-+ (2021).

93. S. Picelli *et al.*, Full-length RNA-seq from single cells using Smart-seq2. *Nat Protoc* **9**, 171-181 (2014).

94. N. L. Bray, H. Pimentel, P. Melsted, L. Pachter, Near-optimal probabilistic RNA-seq quantification. *Nat Biotechnol* **34**, 525-527 (2016).

95. M. I. Love, W. Huber, S. Anders, Moderated estimation of fold change and dispersion for RNA-seq data with DESeq2. *Genome Biol* **15**, (2014).

96. Y. Hao *et al.*, Dictionary learning for integrative, multimodal and scalable single-cell analysis. *Nat Biotechnol* **42**, 293-304 (2024).

97. J. Cao *et al.*, The single-cell transcriptional landscape of mammalian organogenesis. *Nature* **566**, 496-502 (2019).

98. T. Wu *et al.*, clusterProfiler 4.0: A universal enrichment tool for interpreting omics data. *Innovation (Camb)* **2**, 100141 (2021).

99. A. Liberzon *et al.*, The Molecular Signatures Database (MSigDB) hallmark gene set collection. *Cell Syst* **1**, 417-425 (2015).

100. N. D. Bachtel *et al.*, Intestinal mast cell-derived leukotrienes mediate anaphylactic response to ingested antigens. *GitHub*, (2025).


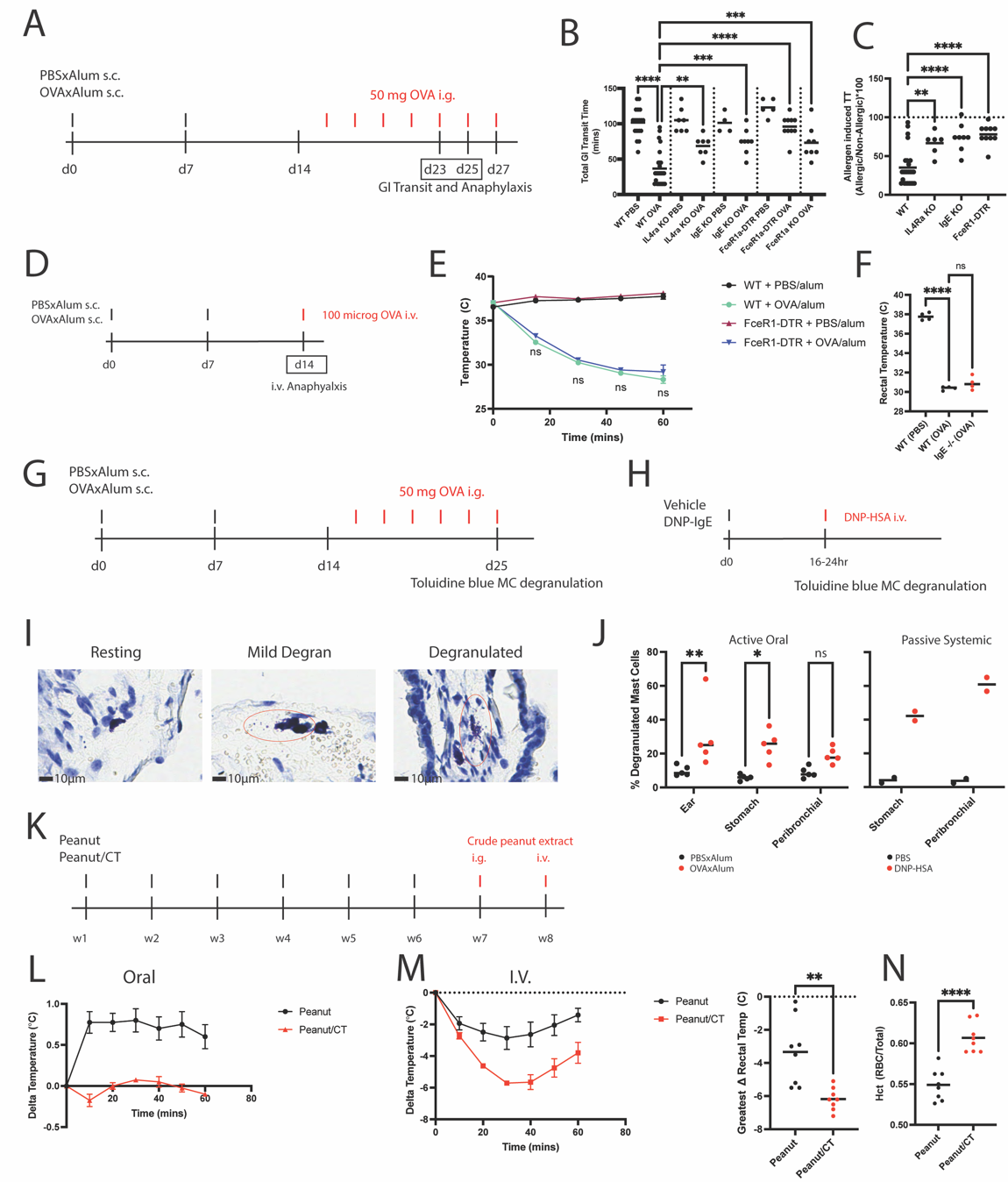


**Fig. S1. FceR1 and non-FceR1 dependent phenotypes and extended analysis of oral anaphylaxis.** (A) BALB/cJ WT, IL4Ra KO, IgE KO, FcεR1 KO, or FcεR1-DTR mice were sensitized subcutaneously with PBSxAlum or OVAxAlum on days 0 and 7, and then challenged with 50mg of OVA 7 times intragastrically . (B) Allergen-inducible gastrointestinal transit time at challenge 5 and (C) GITT expressed as a percentage of the average of non-sensitized controls of each genotype (D) FcεR1-DTR or IgE KO mice were sensitized subcutaneously on d0 and 7 as previously described and challenged intravenously with 100 microg of OVA on d14. (E) Temperature drop over time after i.v. OVA challenge of DT-treated FcεR1-DTR mice. (F) Rectal temperature after 30 minutes of i.v. challenge between sensitized IgE WT or IgE KO mice. Toluidine blue staining was used to assess systemic mast cell degranulation 1 hour after (G) oral challenge or (H) IgE-dependent passive systemic anaphylaxis. (I) representative images of resting and degranulated mast cells. (J) quantification of mast cell degranulation from oral challenge (L) or passive systemic anaphylaxis (R). (K) BALB/cJ mice were sensitized using the peanut/CT model and subsequently challenged intragastrically followed by intravenously. (L) temperature drop to oral challenge or (M) intravenous challenge and (N) hemoconcentration determined at 1hr after intravenous injection. A-C, n=4-28 per group. D-F, n=4-6 per group, I-J, n=2-5 per group, K-N, n=8 per group. At least 2 independent experiments for all panels except for I-J which were one experiment each. Statistics were performed by one way ANOVA with multiple comparisons testing a-j, or unpaired t-test (k-n). Data shows mean +/- SEM. *p<0.05, **p<0.01, ***p<0.001, and ****p<0.0001.


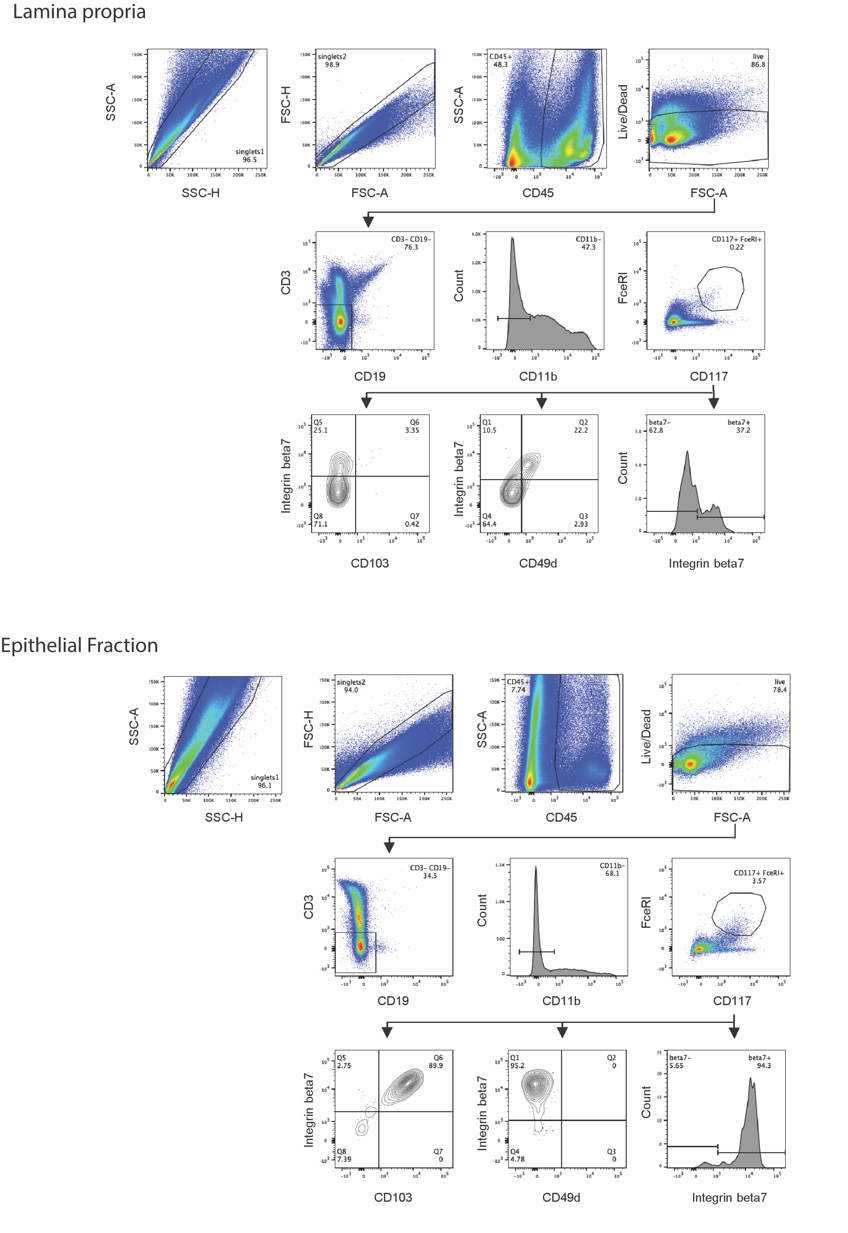


**Fig. S2. Representative flow cytometric gating for intestinal mast cell populations.** Representative flow cytometric gating for quantification of lamina propria or intraepithelial mast cells, and their respective expression of integrin B7, CD103, and CD49d.

**
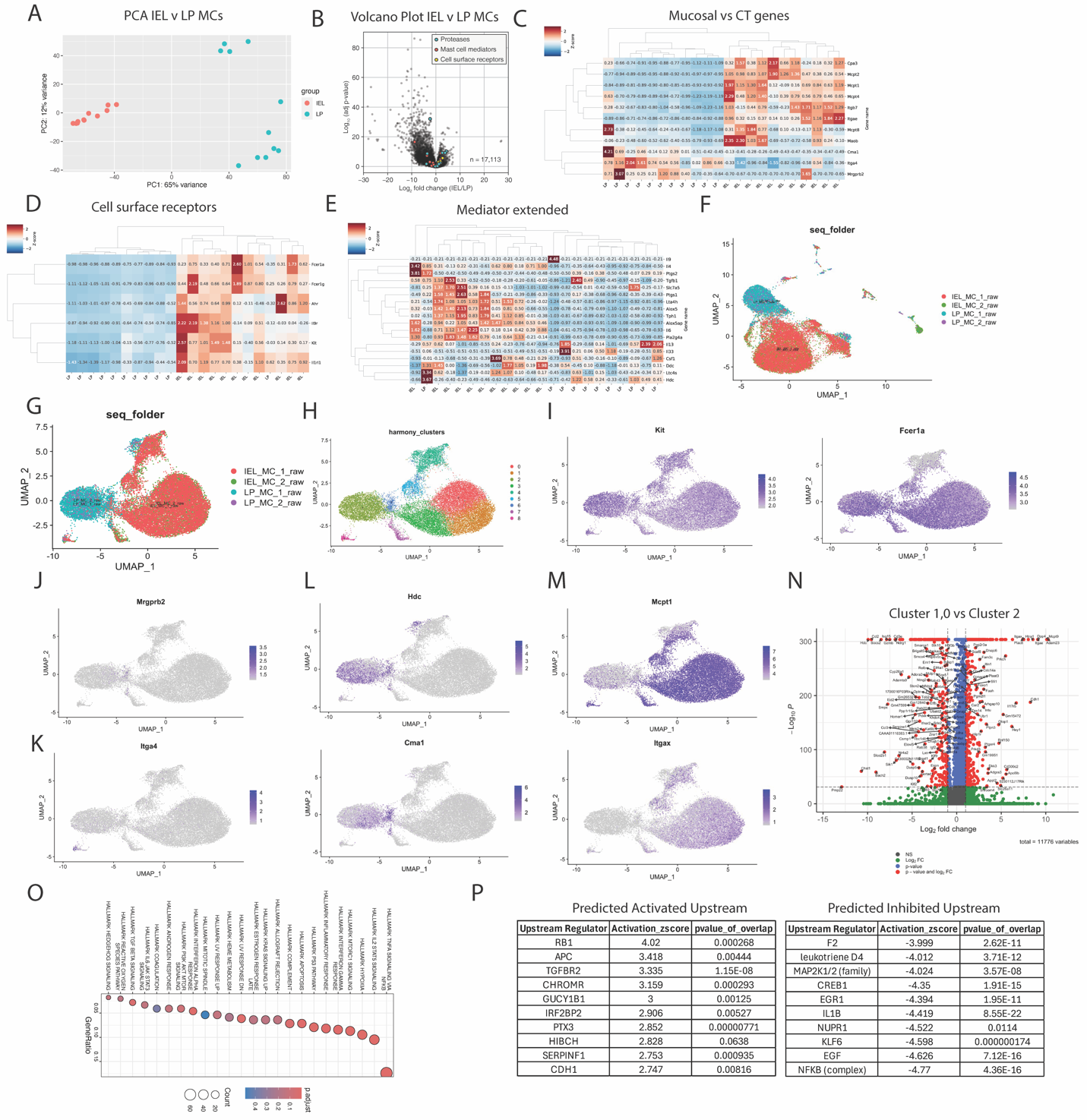
**

**Fig. S3. Extended bulk RNAseq and scRNAseq data analysis of IEL vs LP mast cells.** (A) PCA plot comparing intraepithelial and lamina propria mast cells sorted from sensitized and singly challenged BALBc mice pertaining to Fig 2D and Fig 3B. (B) Volcano plot showing differentially expressed genes between epithelial and lamina propria mast cell populations. Heat map showing well studied (C) mast cell proteases and connective tissue/mucosal mast cell markers, (D) cell surface receptors, and (E) mast cell mediators across each sorted sample. (F) UMAP plot of scRNAseq data from sorted intestinal mast cells with sample identity (LP vs IEL) overlayed (23333 cells were analyzed) pertaining to Fig 2E-I and Fig 3C (G) UMAP plot after non-mast cells were removed (22171 cells were analyzed) (H) harmony clustering of intestinal mast cell samples and expression of (I) *Kit* and *Fcer1a* (J) *Mrgprb2.* Prototypical marker genes for (K) cluster 8, (L) cluster 2, and (M) clusters 0,1. (N) Volcano plot of DEGs between clusters 2 and 0,1 implicated in development of terminally imprinted epithelial MMCs. Red dots showing genes with absolute log2 fold-change > 1 and p value cutoff of 10^-32^. (O) GO term enrichment for DEGs between IEL and LP mast cells and (P) IPA analysis of top 10 predicted upstream activators and suppressors of the IEL mast cell phenotype. For A-E, n=11 samples per compartment from 11 mice, for F-P, n=2 samples per compartment from 2 mice.

**
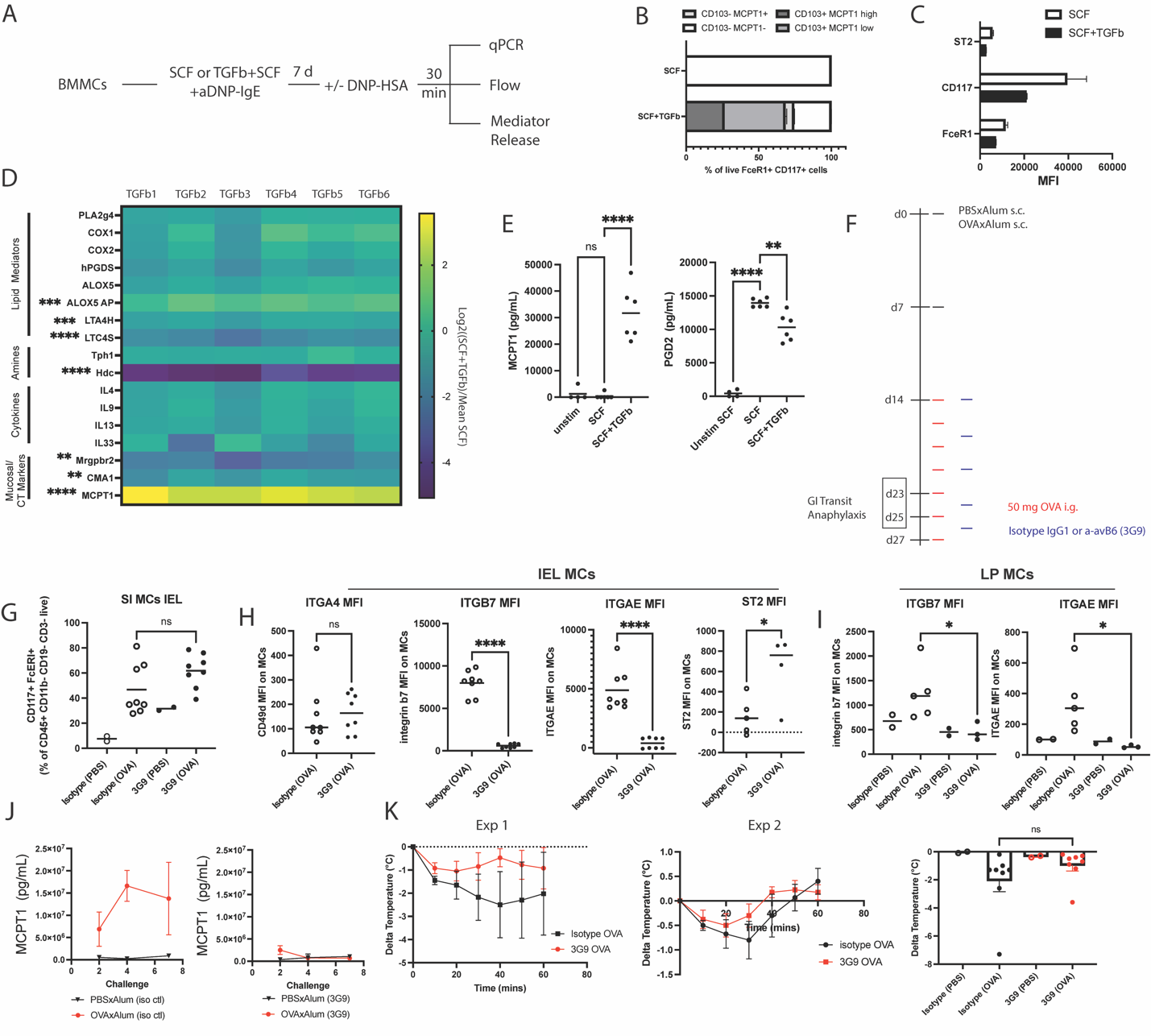
Fig. S4. Roles for TGFb and αvβ6** **in intestinal mast cell imprinting.** (A) Bone marrow mast cells were cultured in the presence of aDNP-IgE, and SCF+/-TGFb for 7 days, degranulated, and analyzed by qPCR, flow cytometry and supernatant ELISA (B) Frequencies of CD103+ MCPT_high_, CD103+ MCPT1_low_, CD103-MCPT1+, and CD103-MCPT1- mast cells elicited by each cytokine condition and (C) ST2, CD117, and FcεR1 MFI. (D) qPCR heatmap for mast cell mediators and connective tissue/mucosal mast cell markers of each TGFb treated sample relative to average values of SCF treated controls. (E) Alterations in MCPT1 and PGD2 elicited by IgE-mediated degranulation of each condition. (F) BALB/cJ female mice were sensitized and orally challenged while being treated with isotype or αvβ6 neutralizing antibody (3G9). (G) numbers of SI mast cells isolated from the intestinal epithelium (H) expression of prototypical mucosal mast cell markers expressed by mast cells from the IEL or (I) LP fractions. (J) serum MCPT1 levels elicited after oral challenge and (K) oral anaphylaxis severity from two independent experimental trials. A-E, n=4-9 independent biological replicates across at least 2 independent experiment. F-K, n=2-8 mice per group across 2 independent experiments. Statistics were performed by unpaired t-test per gene between SCF and SCF+TGFb (D), one way ANOVA with multiple comparisons test (E), or unpaired t-test (G-K). Data shows mean and SEM. *p<0.05, **p<0.01, ***p<0.001, and ****p<0.0001.

**
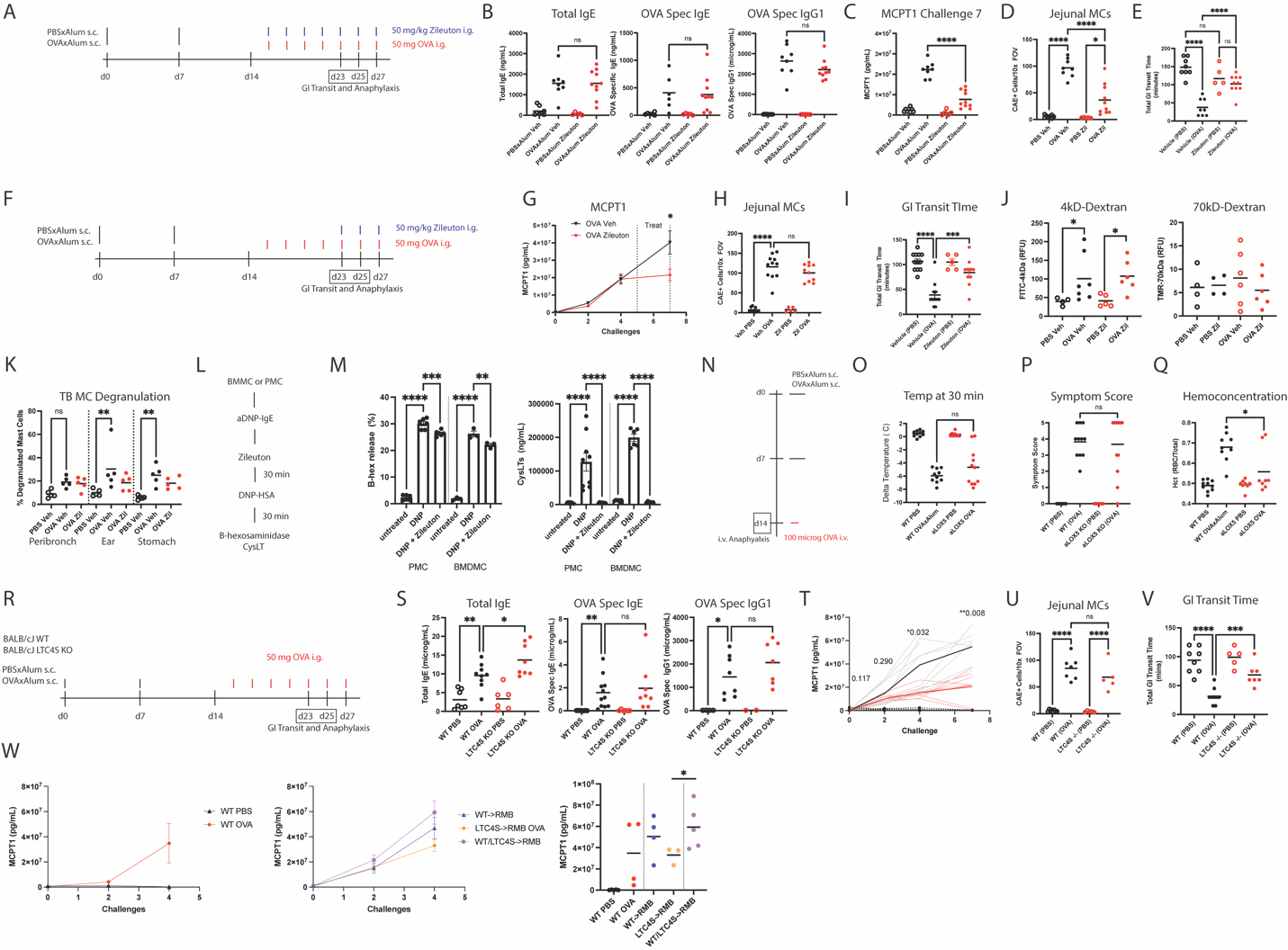
Fig. S5. Extended analysis of aLOX5 blockade and LTC4S KO.** (A) BALB/cJ mice were sensitized subcutaneously with PBSxAlum or OVAxAlum on days 0 and 7 and challenged with 50mg of OVA 7 times intragastrically. Half of the mice received vehicle (black) treatment 1 hour prior to each challenge, and the other received 50mg/kg Zileuton i.g. (red). (B) Serum levels of total IgE, OVA-specific IgE, and OVA-specific IgG1 antibodies at d27. (C) MCPT1 levels at d27 (D) Quantification of jejunal CAE+ mast cell staining /10x FOV. (E) Allergen-induced GITT at 5^th^ OVA challenge by carmine red total GI transit time assay. (F) BALB/cJ mice were sensitized and challenged as above, except treatment began 1 hour prior to 5^th^ challenge. (G) MCPT1 serum levels 1 hour after every other gavage. (H) Jejunal CAE+ mast cells at d27, (I) allergen-inducible GITT (J) intestinal permeability to 4kDa-FITC (L) or 70kDa-TMR (R) dextrans post oral challenge, (K) Zileuton effect on systemic mast cell degranulation by toluidine blue staining, same as Fig S1J with Zileuton as a separate arm of same study, (L) Effect of Zileuton on *in vitro* mast cell degranulation by (M) B-hexosaminidase release and CysLT quantification. (N) C57BL/6J WT or aLOX5 KO mice were sensitized subcutaneously as previously described and challenged intravenously with 100 micrograms of OVA on d14. (O) Temperature drop at 30 minutes after i.v. OVA challenge (P) symptom score and (Q) hematocrit elevation of WT (black) or aLOX5 KO (red) mice. (R) WT BALB/cJ or LTC4S KO mice were sensitized and challenged with i.g. OVA as previously described (S) Serum levels of total IgE, OVA-specific IgE, and OVA-specific IgG1 antibodies at d27. (T) MCPT1 levels over times (U) quantification of CAE+ mast cells per /10x FOV. (V) Total GITT of allergic or control mice at 5^th^ OVA challenge by carmine red total GI transit time assay. (W) MCPT1 serum levels with each oral challenge and at challenge 4 in WT/LTC4S🡪RMB bone marrow chimeras pertaining to Fig 4E. n=3-11 per group for mouse experiments across 2-3 independent experiments except for panels J and K which are each from one. n=3-9 technical replicates across 3 independent experiments for *in vitro* experiments. Statistics were performed by either unpaired t-test (A-C, G, O-Q, T and W) or one way ANOVA with multiple comparisons test (D-E, H-M, S, U-V). Data shows mean +/- SEM. *p<0.05, **p<0.01, ***p<0.001, and ****p<0.0001.

**
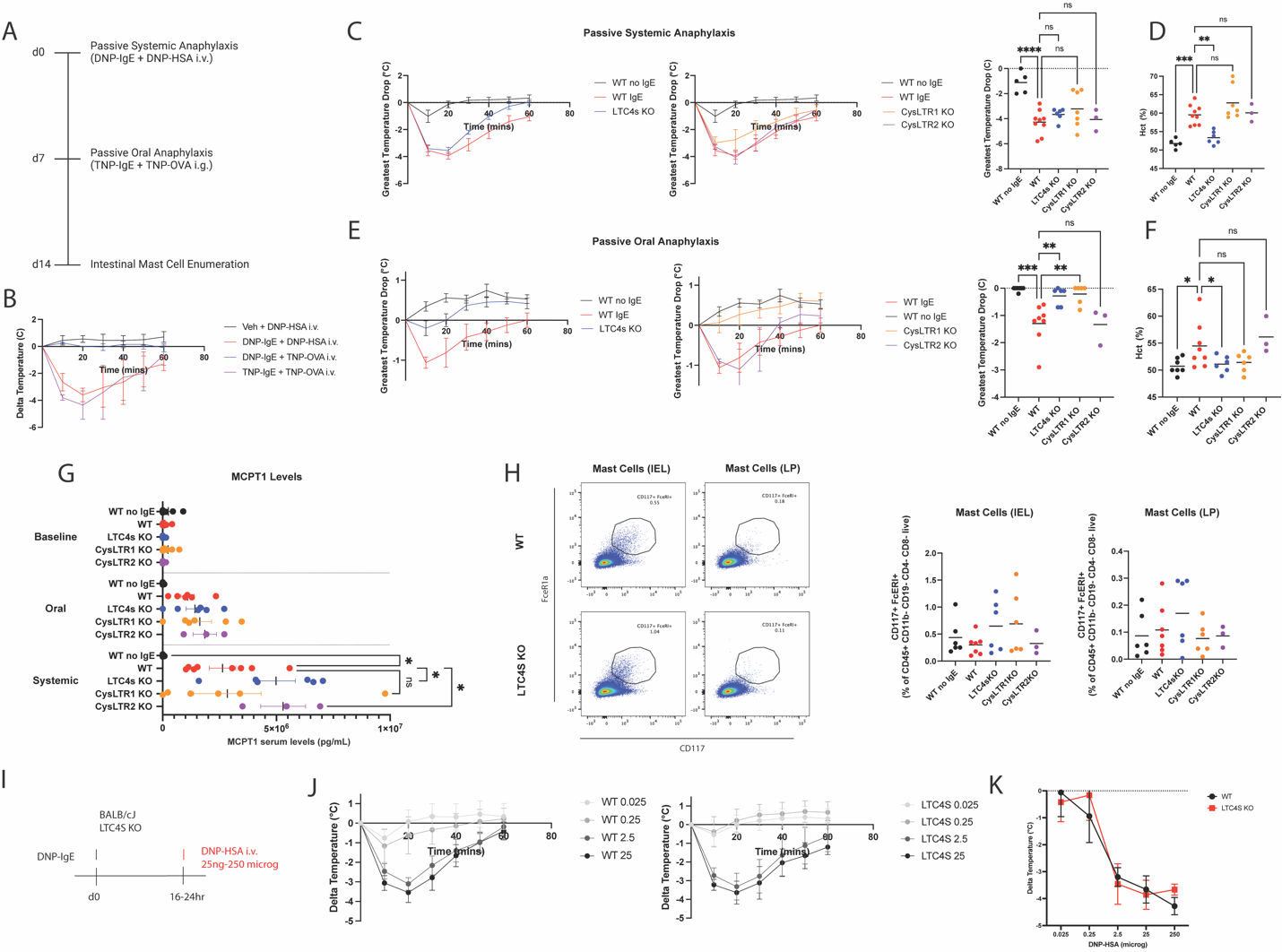
Fig. S6. Leukotrienes have acute route-specific roles in oral anaphylaxis that are unassociated with mucosal mast cell numbers or degranulation.** (A) WT BALB/cJ, LTC4s KO, CysLTR1 KO, and CysLTR2 KO mice were passively administered aDNP-IgE intraperitoneally and challenged 16-24 hours with DNP-HSA i.v. One week later, the same mice were administered aTNP-IgE i.p and then gavaged with 50mg of TNP-OVA i.g. On day 14, mice were harvested and their small intestinal mast cells enumerated by flow cytometry. (B) Temperature drop induced by intravenous administration of antigen and IgE clone combinations (C) greatest temperature drop and (D) hematocrit of leukotriene KO mice following passive systemic challenge. (E) the greatest temperature drop and (F) hematocrit of leukotriene KO mice following passive oral challenge. (G) MCPT1 levels in serum detectible at baseline or 2 hours after oral or intravenous challenge in WT or leukotriene deficient mice (H) representative flow plots of WT and LTC4S KO mast cell staining in SI intraepithelial or lamina propria layers and quantification of MC frequencies in each compartment across mice (I) LTC4S KO or WT mice were passively sensitized and challenged systemically with DNP-HSA of varying doses (J) temperature drop tracings and (K) summary of greatest change in rectal temperature at each challenge dose. A,C-H n=3-9 per group, B n=2 mice per group, I-K n=5-9 mice per concentration pooled from at least 2 independent experiments each except for panel B which was from one. Statistics were performed by one way ANOVA with multiple comparisons test. Each dot represents an individual mouse. Horizontal lines represent mean, error bars represent SEM. *p<0.05, **p<0.01, ***p<0.001, and ****p<0.0001.

**
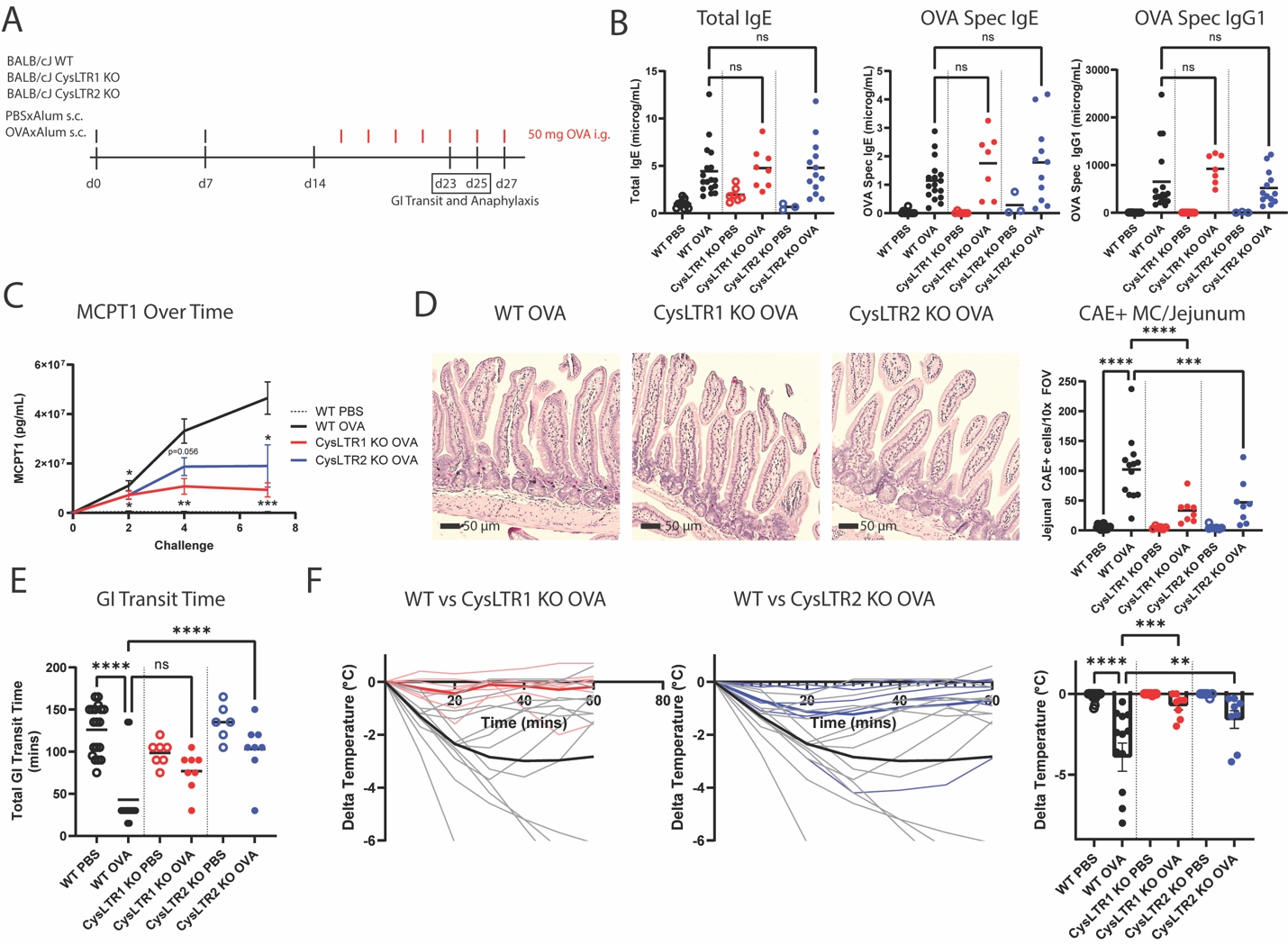
Fig. S7. CysLTR1 and 2 display non-redundant requirements in mucosal mast cell expansion and oral anaphylaxis.** (A) WT BALB/cJ, CysLTR1 KO, or CysLTR2 KO mice were sensitized and challenged with i.g. OVA as previously described (B) Serum levels of Total IgE, OVA-specific IgE antibodies, and OVA-specific IgG1 antibodies at d27. (C) MCPT1 levels over time (D) CAE staining of jejunal segments of control or allergic WT or CysLTR1/2 KO mice and quantification/10x FOV. (E) Total GITT of allergic or control mice at 5^th^ OVA challenge by carmine red total GI transit time assay. (F) Temperature drop over time to 6^th^ i.g. OVA challenge (L) and (R) greatest temperature drop in 1hr. n=6-19 mice per group, pooled from at least 2 independent experiments. Statistics were performed by unpaired t-test (C) or one way ANOVA with multiple comparisons test (B, D-F). Each dot represents an individual mouse. Horizontal lines represent mean, error bars represent SEM. *p<0.05, **p<0.01, ***p<0.001, and ****p<0.0001.

**
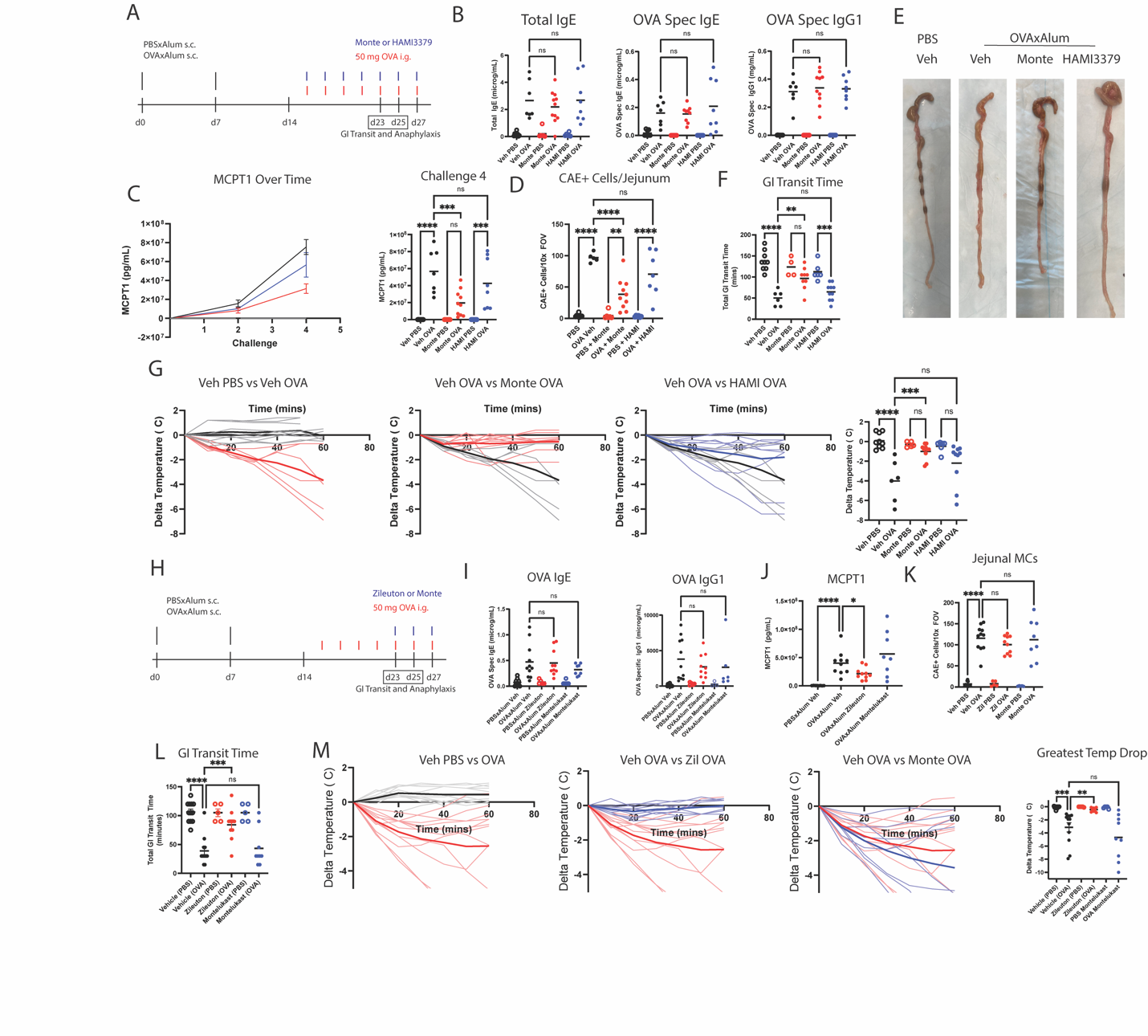
**

**Fig. S8. Chronic but not acute CysLTR1 antagonism prevents mucosal mast cell expansion and oral anaphylaxis.** (A) BALB/cJ mice were sensitized subcutaneously with PBSxAlum or OVAxAlum on days 0 and 7 and then challenged with 50mg of OVA 7 times intragastrically. The mice received vehicle (black), 10mg/kg Montelukast (red) or 0.4mg/kg HAMI3379 intraperitoneally 1 hour prior to each challenge. Total GI transit was measured on 5^th^ challenge, oral anaphylaxis on 6^th^. (B) Serum levels of Total IgE, OVA-Specific IgE, and OVA-Specific IgG1 antibodies at d27. (C) MCPT1 levels over time and at challenge 4 (D) Quantification of jejunal CAE+ mast cell staining /10x FOV. (E) gross colonic inflammation between treatment conditions. (F) Allergen-induced GITT at 5^th^ OVA challenge by carmine red total GI transit time assay. (G) Temperature drop over time at 6^th^ i.g. OVA challenge (L) and (R) greatest temperature drop in 1hr. (H) BALB/cJ mice were sensitized and challenged as previously described, except treatment with vehicle (black), Zileuton 50mg/kg (red), or Montelukast 10mg/kg (blue) began 1 hour prior to 5^th^ challenge. (I) OVA-Specific IgE and IgG1 titres at d27 detectible between different treatment groups. (J) MCPT1 serum levels 1 hour after challenge 7. (K) Jejunal CAE+ mast cells at d27, (L) allergen-induced GITT, and (M) oral anaphylactic temperature drop. Panels A-G, n=4-10 per group across at least two independent experiments. Mice in panels I-M, in vehicle and zileuton groups are the same as in Fig 4B, Fig S5F-I, and montelukast was a separate arm of the same studies. n=5-12. Statistics were performed by one way ANOVA with multiple comparisons test. Data represents mean and SEM. *p<0.05, **p<0.01, ***p<0.001, and ****p<0.0001.


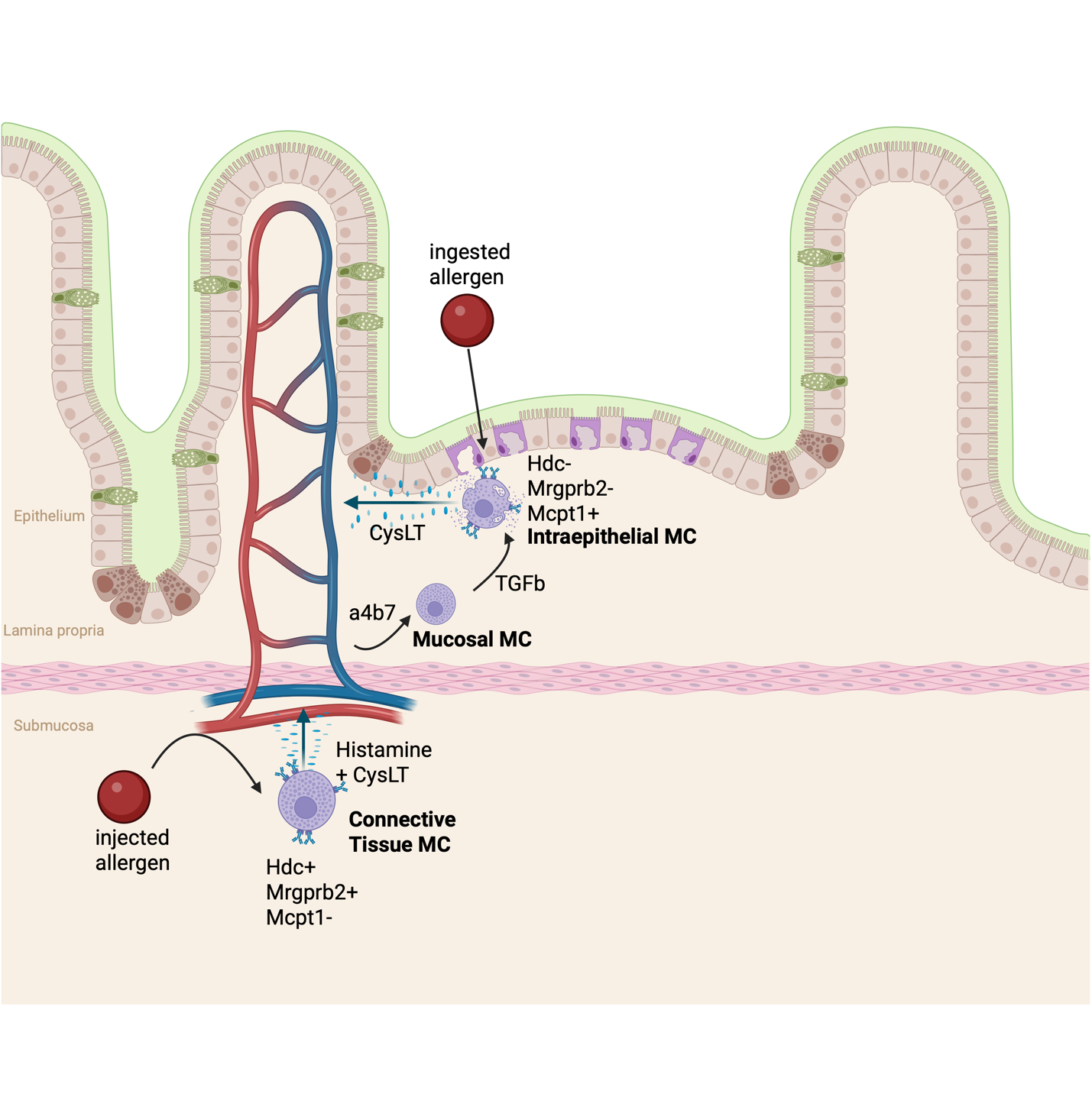


**Fig. S9. Graphical abstract.** TGFb-mediated mucosal mast cell differentiation drives upregulation of MCPT1 and loss of histamine as mast cells home to the epithelium, whereas connective tissue mast cells are histamine rich. Route-dependent requirements for oral anaphylaxis associate with these differences in mediators produced, with cysteinyl leukotrienes selectively required for oral anaphylaxis and histamine necessary for systemic challenge.
